# A thaumatin-like protein of *Ocimum basilicum* confers tolerance to fungal pathogen and abiotic stress in transgenic Arabidopsis

**DOI:** 10.1038/srep25340

**Published:** 2016-05-06

**Authors:** Rajesh Chandra Misra, Sandeep, Mohan Kamthan, Santosh Kumar, Sumit Ghosh

**Affiliations:** 1 Biotechnology Division, Council of Scientific and Industrial Research-Central Institute of Medicinal and Aromatic Plants, Lucknow 226015, India; 2 Council of Scientific and Industrial Research-Indian Institute of Toxicology Research, Lucknow, 226001, India

## Abstract

Plant often responds to fungal pathogens by expressing a group of proteins known as pathogenesis-related proteins (PRs). The expression of PR is mediated through pathogen-induced signal-transduction pathways that are fine-tuned by phytohormones such as methyl jasmonate (MeJA). Here, we report functional characterization of an *Ocimum basilicum* PR5 family member (*ObTLP1*) that was identified from a MeJA-responsive expression sequence tag collection. *ObTLP1* encodes a 226 amino acid polypeptide that showed sequence and structural similarities with a sweet-tasting protein thaumatin of *Thaumatococcus danielli* and also with a stress-responsive protein osmotin of *Nicotiana tabacum*. The expression of *ObTLP1* in *O. basilicum* was found to be organ-preferential under unstressed condition, and responsive to biotic and abiotic stresses, and multiple phytohormone elicitations. Bacterially-expressed recombinant ObTLP1 inhibited mycelial growth of the phytopathogenic fungi, *Scleretonia sclerotiorum* and *Botrytis cinerea*; thereby, suggesting its antifungal activity. Ectopic expression of *ObTLP1* in Arabidopsis led to enhanced tolerance to *S. sclerotiorum* and *B. cinerea* infections, and also to dehydration and salt stress. Moreover, induced expression of the defense marker genes suggested up-regulation of the defense-response pathways in *ObTLP1*-expressing Arabidopsis upon fungal challenge. Thus, *ObTLP1* might be useful for providing tolerance to the fungal pathogens and abiotic stresses in crops.

Because of their sessile nature, plants often exposed to diverse biotic and abiotic stress conditions throughout their life cycle. To counteract these environmental constrains, plants have evolved highly complex and sophisticated defense mechanisms^1^,^2^. In case of attacks by phytopathogenic fungi, the defense responses include reactive oxygen species (ROS)-triggered localized cell death i.e. hypersensitive response (HR) at the infection site, accumulation of the antimicrobial phytochemicals (phytoalexins) and expression of a group of proteins known as pathogenesis-related (PR) proteins^3^,^4^. PRs are classified into 17 families (PR1 to PR17) based on their amino acid composition, structure and biochemical function^5^. Among these, PR5 members show striking sequence similarity with thaumatin^6^, an intensively sweet-tasting protein of West African shrub *Thaumatococcus danielli* and therefore, also known as thaumatin-like proteins (TLPs). In general, TLPs are low molecular-weight (20–26 kDa) proteins with sixteen conserved cysteine (Cys) residues. Cys residues are involved in mediating eight intra-molecular disulfide bonds which are suggested to provide stability to the proteins under extreme pH and temperature ranges^7^. The expressions of TLPs have been shown to up-regulate when plants are exposed to biotic and abiotic stress conditions^8^,^9^. Moreover, some TLPs have been shown to exhibit antifungal activity and over-expressed in plants to test their effectiveness to provide tolerance to fungal pathogens^9^,^10^,^11^,^12^,^13^,^14^. However, the molecular mechanism for the antifungal activity is still not clear and the biological function of TLPs in plants is yet to be established.

Previous studies suggested that the antifungal property of TLPs may be attributed to the inhibition of activities of the fungal enzymes such as β -glucanase, xylanase, α -amylase and trypsin, and also due to the ability to rupture fungal membrane by pore formation^15^,^16^,^17^,^18^,^19^. Moreover, TLP may also act by inducing programmed cell death in fungi following binding to the plasma membrane receptor protein that functions in the RAS2/cAMP signalling pathway^20^,^21^. Besides providing defense against pathogenic fungi, TLPs might also help plants to mitigate various abiotic stresses^9^,^18^,^22^,^23^. Moreover, TLPs are also recognised as osmotin-like proteins (OLPs) based on their sequence homology with the osmotin that accumulates at high levels when cells begin to adapt to a low water potential environment^24^,^25^.

Phytohormones methyl jasmonate (MeJA), ethylene (ET) and salicylic acid (SA) are the primary mediators of plant defense response towards attack by fungal pathogens^2^. Defense response mediated by SA is known to trigger resistance against biotrophic and hemibiotrophic pathogens. However, combined effect of JA and ET-mediated defense responses provide resistance against necrotrophic pathogens^26^. Moreover, abscisic acid (ABA) can act as a positive or negative regulator of plant defense response depending on nature of the pathogens^27^,^28^. The expression of PRs has been shown to induce when plants are externally treated with phytohormones or exposed to different biotic and abiotic stress conditions^8^,^9^,^29^,^30^. Therefore, transcriptional profiling of plant’s response to phytohormone treatment is an important strategy for the identification of PRs involved in plant defense response. Previously, we have reported a collection of *Ocimum basilicum* transcripts that are MeJA inducible, including transcripts with sequence similarity with the *PR5s* and *PR10s* of other plant species^31^. Here, we report detailed analysis and functional characterization of an *O. basilicum* PR5 family member (*ObTLP1*) following bacterial and plant expressions. Bacterially-expressed recombinant ObTLP1 showed antifungal activity towards phytopathogenic fungi *Scleretonia sclerotiorum* and *Botrytis cinerea*. Moreover, ectopic expression of *ObTLP1* in *Arabidopsis thaliana* led to enhanced tolerance to the fungal pathogens and also to abiotic stresses.

## Results

### Identification and expression profiling of *ObTLP1*

**Figure 1:**
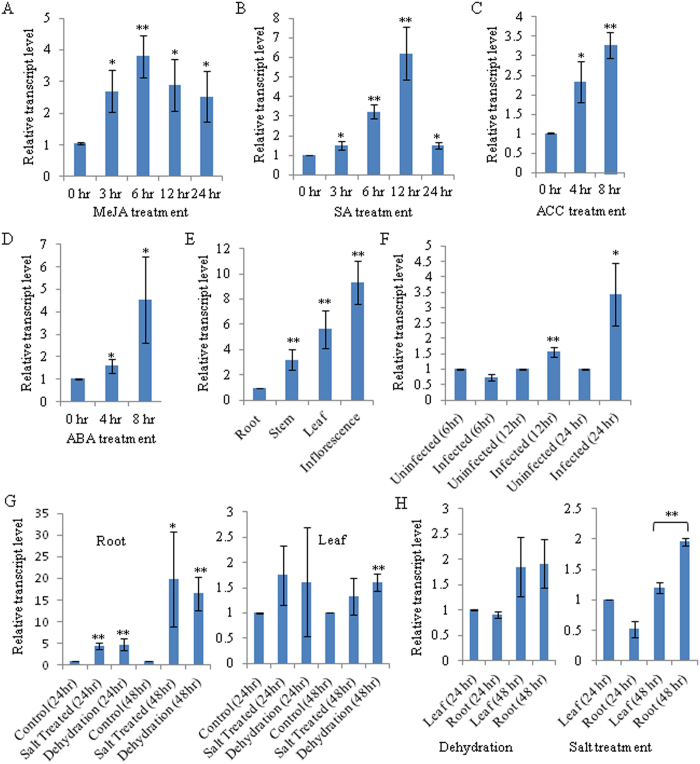
*ObTLP1* transcript expression pattern in *O. basilicum*. (**A–D**) MeJA, SA, ET and ABA-inducible expression of *ObTLP1* was determined by qRT-PCR analysis. (**E**) Transcript expression pattern of *ObTLP1* in different plant organs was determined by qRT-PCR. (**F,G**) *ObTLP1* transcript expression in leaves after *S. sclerotiorum* infection (**F**), and in roots and leaves after dehydration and salt stress (**G**), was determined by qRT-PCR. (**H**) Relative transcript level of *ObTLP1* in roots and leaves after dehydration and salt stress. Data are mean ±  s.d. (n =  3 to 4). Asterisks indicate statistically significant difference at **P <  0.01 and *p <  0.05 using two-tailed Student’s t-test.

An expression sequence tag (EST) library, previously prepared from the MeJA-elicited *O. basilicum*^31^, was screened for the nucleotide sequences with a homology to the members of different PR families. This resulted in the identification of a 575 bp EST (JZ190528) with homology to the PR5 family members, in addition to three ESTs with homology to the PR10 family members. Among these PRs, PR5 was further considered for the detailed functional analysis. A full-length complementary DNA (cDNA) sequence specific to the PR5 was obtained from *O. basilicum* leaf total RNA by a combination of reverse transcription-polymerase chain reaction (RT-PCR) amplification of the EST sequence and rapid amplification of the cDNA end (3′ RACE). The complete cDNA sequence (725 bp) was submitted to the GenBank database under the accession number JQ793640 and designated as *ObTLP1* (*O. basilicum thaumatin-like protein 1*). In order to confirm MeJA induced expression, quantitative RT-PCR (qRT-PCR) was employed to determine *ObTLP1* transcript level in MeJA-treated *O. basilicum*. The analysis revealed increased transcript level of the *ObTLP1* in MeJA-treated *O. basilicum* compared to the control, at 3 hr to 24 hr after treatment with 250 μ M MeJA (Fig. 1A). Further to test whether *ObTLP1* is also responsive to other phytohormone elicitations, the transcript expression pattern of the *ObTLP1* was determined in *O. basilicum* after treatments with SA, ABA and 1-aminocyclopropane-1-carboxylic acid (ACC), a precursor of the ET (Fig. 1B–D). Moreover, *ObTLP1* transcript level was also examined under dehydration and salt stress (Fig. 1G,H), and after infection with *S. sclerotiorum* (Fig. 1F) which was earlier reported to be a fungal pathogen of *O. basilicum*^32^,^33^. The transcriptional upregulation of *ObTLP1* after treatments with MeJA, SA, ABA and ACC, and also under abiotic and biotic stresses suggested that *ObTLP1* is responsive to multiple phytohormone and stress-mediated signal transduction pathways. These results corroborate with earlier findings that suggested the role of PR5 family members in providing defense to plants against multiple stresses^9^,^18^. Further, *ObTLP1* expression patterns in different plant organs were determined by qRT-PCR analysis (Fig. 1E). *ObTLP1* transcript was detected in all plant parts including roots, stems, leaves and inflorescences. However, the transcript level was more in upper ground parts of the plant compared to the roots and highest transcript expression was recorded in inflorescences. This organ-preferential transcript expression pattern suggested a possible biological role for *ObTLP1* in protecting the upper ground parts of the plants, particularly inflorescences, from fungal infection.

### ObTLP1 sequence and structural analysis

Sequence analysis revealed that *ObTLP1* encodes for a 226 amino acids polypeptide with a calculated molecular mass of 24.95 kDa and a theoretical pI of 8.11 (http://web.expasy.org/). An N-terminal signal peptide of 23 amino acids was recognised in ObTLP1 following TargetP 1.1 and SignalP 4.1 servers (http://www.cbs.dtu.dk/), suggested it to be a secretory protein that is possibly localized to the extracellular space as determined by YLoc+ (http://abi.inf.uni-tuebingen.de/Services/YLoc/webloc.cgi). At the amino acid sequence level, ObTLP1 shared 98.67% identity (ClustalW score, http://www.ebi.ac.uk/) with a recently reported antifungal protein of *O. basilicum* (ObOLP) whose *in planta* function and antifungal activity against phytopathogenic fungi are yet to be determined^34^. As compared to ObTLP1, ObOLP is one amino acid shorter with a deletion of leucine residue within a repetitive sequence at the N-terminal end and also has three amino acid substitutions (Supplementary Fig. S1). These discrepancies between ObTLP1 and ObOLP at the amino acid sequence level may be due to different cultivars from where they were isolated. However, it is also possible that *ObTLP1* and *ObOLP* are two separate genes of *O. basilicum* that were resulted from a gene duplication event. Beside this, ObTLP1 shared 59.9% and 55.56% sequence identities with *Glycine max* and *Vitis vinifera* TLPs, respectively, and 50.88% sequence identity with *Nicotiana tabacum* osmotin, a stress-responsive protein^25^ (Supplementary Fig. S1).

**Figure 2:**
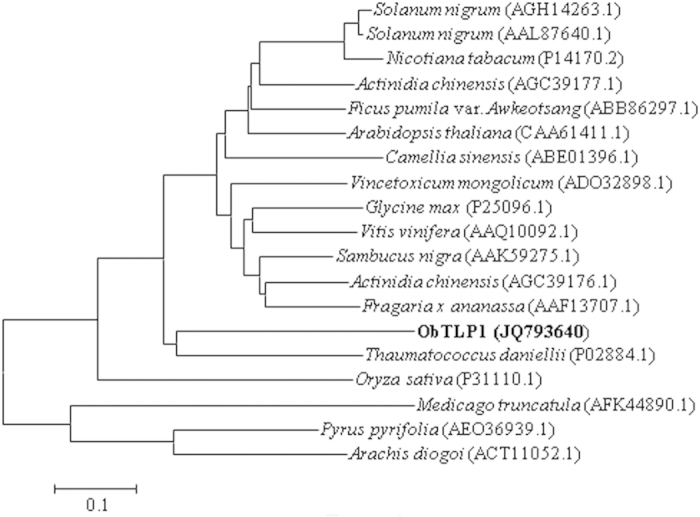
Phylogenetic tree depicting evolutionary relationships of TLP orthologues. The complete amino acid sequences of TLPs obtained from the GenBank database, together with that of ObTLP1 were analyzed through MEGA6 using the Neighbor-Joining Method. The evolutionary distances were computed using the Poisson correction method and are in the units of the number of amino acid substitutions per site.

**Figure 3:**
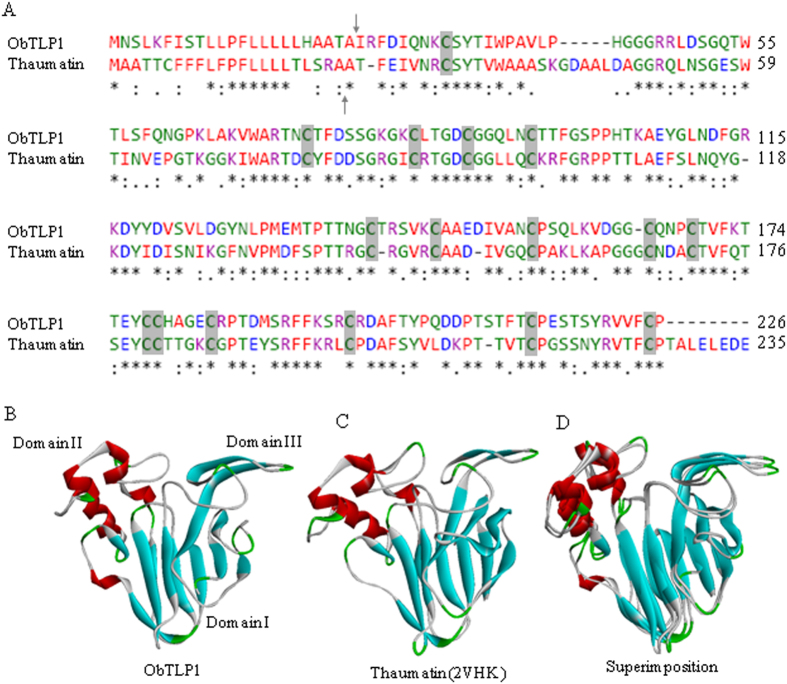
Sequence and structural similarities between ObTLP1 and thaumatin. (**A**) Pair-wise alignment of amino acid sequences of ObTLP1 and thaumatin. Putative cleavage sites for signal peptides are marked with arrows. Identical amino acid residues are depicted as *, while conserved and semi-conserved substitutions are marked as : and ., respectively. Conserved cystine residues are highlighted. (**B**) Predicted 3D structure of ObTLP1 based on homology modeling. (**C**) Crystal structure of thaumatin (2VHK) was used as the template for homology modeling. (**D**) Superimposition of 3D structures of ObTLP1 and thaumatin.

A phylogenetic tree was constructed to depict the relatedness of ObTLP1 with the characterized TLPs from other plant species (Fig. 2). In the phylogenetic tree, ObTLP1 was grouped with the thaumatin^6^ of *T. danielli*; suggesting that ObTLP1 and thaumatin are closely related. Further, to examine similarities between ObTLP1 and thaumatin, their amino acid sequences and three dimensional (3D) structures were compared (Fig. 3A–D). At the amino acid sequence level, ObTLP1 shared 54.67% identity (ClustalW score, http://www.ebi.ac.uk/) with thaumatin (P02884). Similar to thaumatin, ObTLP1 contains sixteen cysteine residues that are also conserved in other TLPs (Supplementary Fig. S1). The involvement of these cysteine residues for the formation of eight intramolecular disulphide bonds in ObTLP1 was predicted (Supplementary Fig. S2). To get an idea about the 3D structure of ObTLP1, homology modeling was carried out using a crystal structure (0.94 Å atomic resolution) of thaumatin (2VHK) as template^35^. The 3D structural model of ObTLP1 was quite similar to 2VHK and three conserved domains (domain I to III)^36^ were revealed (Fig. 3B–D). These results suggested that *ObTLP1* encodes a thaumatin-like protein that might function in stress response.

### Antifungal activity of the bacterially-expressed ObTLP1

**Figure 4:**
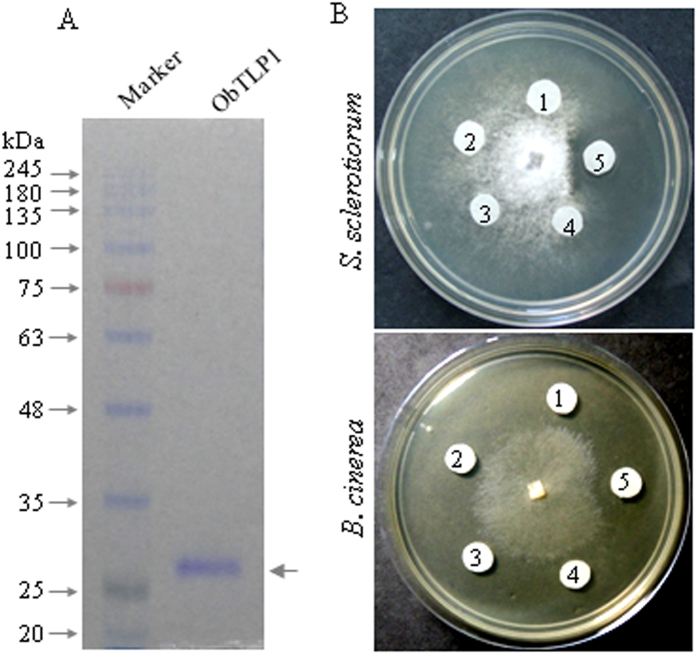
ObTLP1 is an antifungal protein. (**A**) Bacterially-expressed N-terminally polyhistidine-tagged ObTLP1 was resolved as ~28 kDa protein on 10% SDS-PAGE. (**B**) Purified ObTLP1 inhibited mycelial growth of phytopathogenic fungi *S. sclerotiorum* and *B. cinerea* in agar disc diffusion assays. 1- buffer only; 2- BSA in buffer; 3 to 5- 3 μ g, 10 μ g and 30 μ g of purified ObTLP1 in 40 μ l buffer, respectively. Photographs were taken 24 hr post incubation.

ObTLP1 showed sequence similarity to the antifungal proteins of other plant species^9^,^11^,^37^. Therefore, to perform *in vitro* antifungal assays, ObTLP1 was expressed and purified from *Escherichia coli* (BL21-CodonPlus (DE3)-RIPL strain) as N-terminal 6xHis-tagged proteins (Fig. 4A). Analysis of soluble and insoluble protein fractions of 0.2 mM isopropyl thiogalactopyranoside (IPTG)-induced bacterial culture grown at 18 ^o^C revealed low level expression of ObTLP1 as a soluble protein. Recombinant ObTLP1 was purified from soluble fraction by using a nickel-nitrilotriacetic acid (Ni-NTA) agarose column as described in Methods. Purified protein was resolved as a ~28 kDa polypeptide in 10% SDS-PAGE gel (Fig. 4A).

To test antifungal activity of ObTLP1, fungal growth inhibition was determined following agar disc diffusion method as carried out previously^9^. For this, we selected phytopathogenic fungi *S. sclerotiorum* and *B. cinerea* that cause substantial loss in global crop yields by infecting a range of plant species^38^,^39^. Various concentrations of ObTLP1 (3–30 μ g in 40 μ l buffer) were added to the blank Whatman filter paper discs evenly placed on agar plate around a fungal colony grown for one (*S. sclerotiorum*) or two days (*B. cinerea*), and plates were incubated for 24 hr at 23 ^o^C. For control experiment, only buffer and bovine serum albumin (BSA) prepared in buffer were added to the blank Whatman filter paper discs instead of the purified ObTLP1. Examination of the spreading colonies of *S. sclerotiorum* and *B. cinerea* at 24 hr after incubation revealed a clear zone of inhibition around the Whatman filter paper discs added with 30 μg of purified ObTLP1 (Fig. 4B). However, growth inhibition was not observed when concentrations of ObTLP1 were either low (≤ 0.25 μ g/μ l) or Whatman filter paper disc contained BSA or only buffer. These results clearly indicated a dose-dependent antifungal activity of ObTLP1 against phytopathogenic fungi.

### Ectopic expression of *ObTLP1* in Arabidopsis led to enhanced tolerance to the fungal pathogens, *S. sclerotiorum* and *B. cinerea*

**Figure 5:**
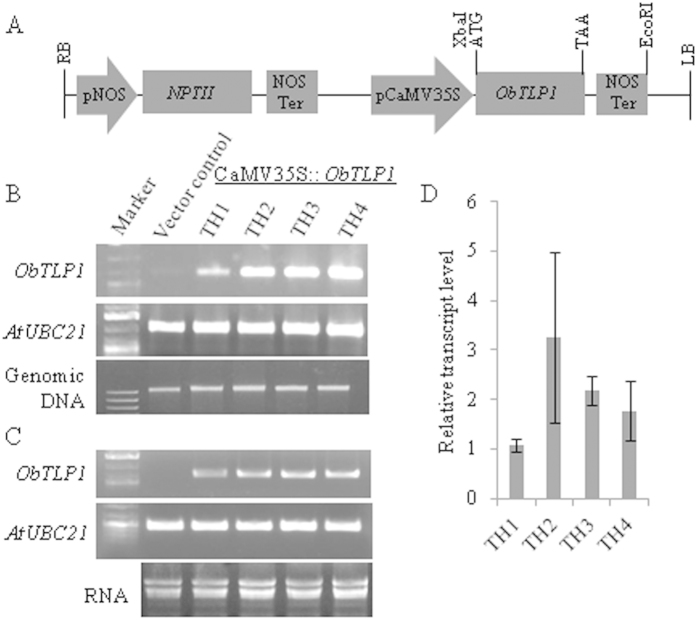
CaMV35S promoter-driven expression of *ObTLP1* in Arabidopsis. (**A**) Schematic representation of CaMV35S::*ObTLP1* construct used for *Agrobacterium*-mediated transformation of Arabidopsis following floral dip method. (**B**) Conformation of the genomic integration of CaMV35S::*ObTLP1* construct into independent transgenic lines (TH1-TH4) following PCR analysis using genomic DNA as template. (**C**) sqRT-PCR analysis confirmed transcript expression of *ObTLP1* in leaves of independent transgenic lines (TH1-TH4). *AtUBC21* (AT5G25760) amplifications (1082 bp and 448 bp for genomic and cDNA, respectively) were considered as PCR control. *ObTLP1* and *AtUBC21*gels have been run under the same experimental conditions. Full-length gels are presented in Supplementary Fig. S7. (**D**) Relative transcript level of *ObTLP1* in leaves of transgenic lines was determined following qRT-PCR analysis. Data are mean ±  s.d. from three biological replicates.

To test *in planta* function, *ObTLP1* was constitutively expressed in Arabidopsis under the control of CaMV35S promoter (Fig. 5A). A total of four independent transgenic lines were developed and grown for four successive generations for selecting homozygous lines. Stable genomic integration of CaMV35S::*ObTLP1* construct was tested in T5 homozygous transgenic lines following PCR using genomic DNA as template (Fig. 5B). The results revealed stable genomic integration of CaMV35S::*ObTLP1* construct in all the transgenic lines. The transcript expression of *ObTLP1* in T5 homozygous transgenic lines was also confirmed following sqRT-PCR analysis (Fig. 5C). The relative transcript level of *ObTLP1* in different transgenic lines, determined by qRT-PCR, revealed highest transcript expression in TH2 line (Fig. 5D). In rest of the transgenic lines, *ObTLP1* expression level was quite comparable. Further, two *ObTLP1*-expressing homozygous independent transgenic lines (TH1 and TH2) with single copy transgene integration, were considered for the detailed phenotypic characterization (Supplementary Fig. S3D). Transgenic lines were indistinguishable from vector control plants (transformed with empty vector pBI121) with respect to their growth, development and reproduction (Supplementary Table S1; Supplementary Fig. S3A,B). Arabidopsis genome encodes an ortholog of ObTLP1 (Fig. 2). Thus, we were interested to know whether the expression of Arabidopsis TLP (AtOSM34, AT4G11650) was affected due to the ectopic expression of *ObTLP1*. However, *AtOSM34* transcript expression was found to be unaltered in *ObTLP1*-expressing transgenic lines (Supplementary Fig. S3C).

**Figure 6:**
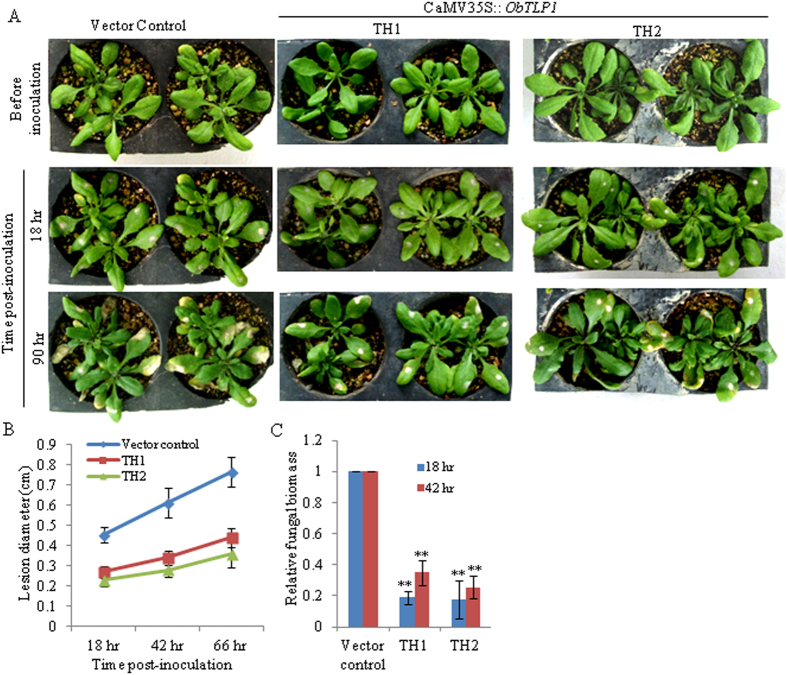
*ObTLP1*-expressing Arabidopsis plants are tolerant to *S. sclerotiorum* infection. (**A**) Leaves of four-week-old *ObTLP1*-expressing transgenic lines (TH1 and TH2) and vector control plants were inoculated with equal amount of mycelial suspension of *S. sclerotiorum* as described in Methods. Disease assessment was carried out at different time intervals and photographs were taken. (**B**) Lesion size was measured at different time intervals in infected leaves. Data are mean ±  s.d. from sixteen plants for each transgenic line. (**C**) Fungal biomass in infected leaves was quantified by qPCR. Relative level of *S. sclerotiorum* ITS genomic DNA was determined using Arabidopsis chloroplast-encoded ribulose-1,5-bis-phosphate carboxylase/oxygenase large subunit as reference gene. Data are mean ±  s.d. from three biological replicates. Asterisks indicate statistically significant difference at **P <  0.01 using two-tailed Student’s t-test.

**Figure 7:**
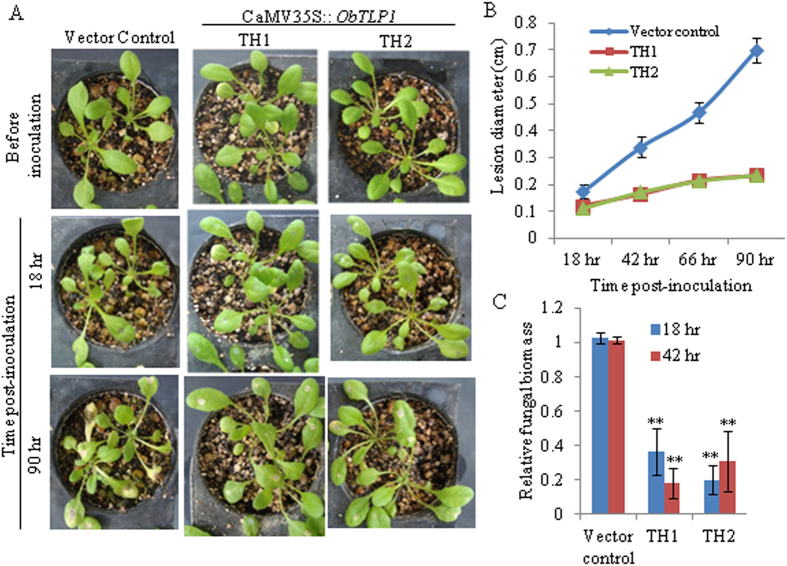
*ObTLP1*-expressing Arabidopsis plants are tolerant to *B. cinerea* infection. (**A**) Leaves of four-week-old *ObTLP1*-expressing transgenic lines (TH1 and TH2) and vector control plants were inoculated with equal amount of spore suspension of *B. cinerea* as described in Methods. Disease assessment was carried out at different time intervals and photographs were taken. (**B**) Lesion size was measured at different time intervals in infected leaves. Data are mean ±  s.d. from sixteen (vector control) or eight (*ObTLP1*-expressing lines) plants. (**C**) Fungal biomass in infected leaves was quantified by qPCR. Relative level of *B. cinerea Actin* genomic DNA was determined using Arabidopsis chloroplast-encoded ribulose-1,5-bis-phosphate carboxylase/oxygenase large subunit as reference gene. Data are mean ±  s.d. from three biological replicates. Asterisks indicate statistically significant difference at **P <  0.01 using two-tailed Student’s t-test.

To determine whether *ObTLP1* expression can provide fungal tolerance, leaves of four-week-old T5 homozygous transgenic lines and vector control plants were inoculated with mycelial suspension of *S. sclerotiorum* as described in Methods, and disease development was recorded for up to 90 hour post-inoculation (hpi). As compared to the vector control plants, TH1 and TH2 transgenic lines showed delayed disease development and slow spreading of the necrotic lesions around the infection site (Fig. 6A,B). Moreover, fungal growth in infected leaves, as determined by qPCR analysis of *S. sclerotiorum* genomic DNA level, was less in TH1 and TH2 transgenic lines as compared to the vector control plants (Fig. 6C). Similarly, TH1 and TH2 transgenic lines were also tolerant to *B. cinerea* infection (Fig. 7A–C). These results clearly demonstrated enhanced tolerance of the Arabidopsis towards fungal pathogens following ectopic expression of *ObTLP1*.

### Expression analysis of the defense marker genes in *ObTLP1*-expressing Arabidopsis

**Figure 8:**
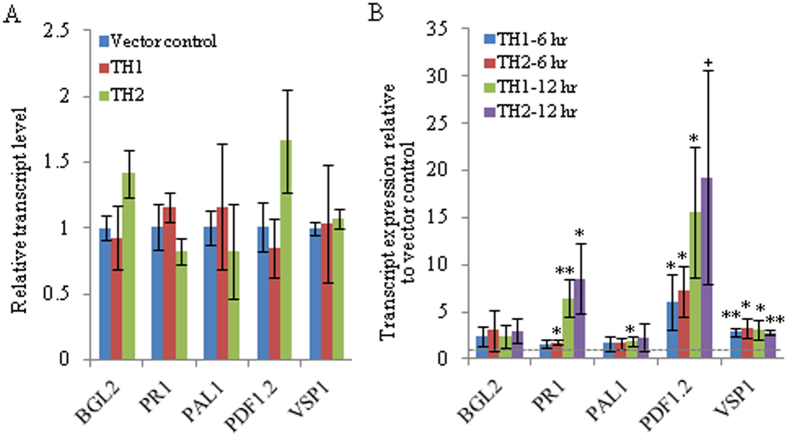
Expression patterns of the defense marker genes in *ObTLP1*-expressing Arabidopsis. (**A**) Relative transcript level was determined in uninfected leaves. Data are mean ±  s.d. (n =  4, from two biological samples). (**B**) Relative transcript level was determined in *S. sclerotiorum* infected leaves. Data are presented as fold expression relative to the vector control for each time points. Data are mean ±  s.d. from three biological replicates. Asterisks indicate statistically significant difference at **P <  0.01 and *p <  0.05 using two-tailed Student’s t-test. ‘+’ denotes P value of 0.051.

To investigate whether enhanced tolerance to the fungal pathogens was due to the altered defense response, the expression patterns of the defense marker genes was compared in vector control and *ObTLP1*-expressing Arabidopsis lines (Fig. 8A,B). The transcript expression patterns of the Arabidopsis *PATHOGENESIS-RELATED PROTEIN 1* (*PR1*), *PHENYLALANINE AMMONIA-LYASE* (*PAL1*), *β*-*1,3-GLUCANASE2* (*BGL2*), *PLANT DEFENSIN PROTEIN1.2* (*PDF1.2*) and *VEGETATIVE STORAGE PROTEIN1* (*VSP1*) that are well know markers for either SA (PR1, PAL1 and BGL2) or JA/ET (PDF1.2 and VSP1)-mediated defense pathways^40^, were determined in vector control and *ObTLP1*-expressing Arabidopsis lines under standard growth condition (Fig. 8A), and also after *S. sclerotiorum* infection (Fig. 8B). Under standard growth condition, the expression level of these defence genes was similar in vector control and *ObTLP1*-expressing Arabidopsis (considering both TH1 and TH2 lines). However, when expression pattern of these genes was compared at 6 hpi and 12 hpi with *S. sclerotiorum*, a marked increase in *PDF1.2* transcript level was noticed in *ObTLP1*-expressing lines compared to the vector control. Moreover, a significant increase in *VSP1* transcript level was also detected in *ObTLP1*-expressing lines. However, at 12 hpi with *S. sclerotiorum*, *PR1* transcript was detected at significantly higher level in both the *ObTLP1*-expressing lines compared to the vector control. This transcript expression patterns suggested an early induction of the JA/ET-mediated defense in *ObTLP1*-expressing lines after *S. sclerotiorum* infection compared to the SA-mediated defense.

### ObTLP1-expressing Arabidopsis are tolerant to the abiotic stresses

**Figure 9:**
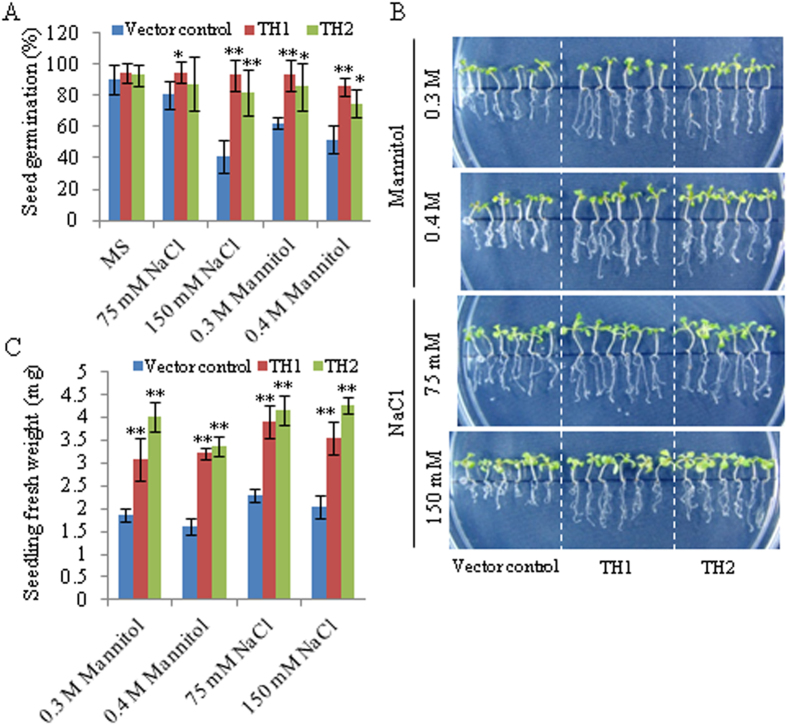
*ObTLP1*-expressing Arabidopsis plants are tolerant to abiotic stresses. (**A**) Germination rate of the *ObTLP1*-expressing transgenic lines (TH1 and TH2), and vector control plants was determined under dehydration (mannitol) and salt (NaCl) stress at 8 days of incubation after stratification. Germination rates (%) are presented as mean ±  s.d., obtained from at least four plates. (**B**) Growth of the TH1 and TH2 lines, and vector control plants under dehydration and salt stress. Seedlings, after 5 days of germination on MS, were placed onto MS +  mannitol/NaCl plates, and grown for additional 10 days. Photographs of the seedlings obtained from 10 days of growth are shown. (**C**) Seedlings fresh weight represents mean ±  s.d. (n =  18). Asterisks indicate statistically significant difference at **P <  0.01 and *p <  0.05 using two-tailed Student’s t-test.

As *ObTLP1* transcript level was upregulated under abiotic stress conditions (Fig. 1G,H) and also in response to multiple phytohormone elicitations, including abiotic stress regulator ABA (Fig. 1A–D), we next tested whether *ObTLP1* could be involved in mediating abiotic stress responses in transgenic Arabidopsis. For this, seed germination (Fig. 9A) and seedling growth (Fig. 9B,C) of the vector control and *ObTLP1*-expressing Arabidopsis lines were determined under dehydration (0.3 M and 0.4 M mannitol) and salt (75 mM and 150 mM NaCl) stress conditions. Vector control and *ObTLP1*-expressing Arabidopsis lines displayed similar germination rates under the normal growth condition (Fig. 9A), suggesting that expression of *ObTLP1* did not affect seed germination under unstressed condition. However, in the presence of NaCl and mannitol, *ObTLP1*-expressing Arabidopsis lines exhibited higher seed germination rate compared to the vector control (Fig. 9A). In the presence of 150 mM NaCl, 92% and 82% seed germination rates were observed for the TH1 and TH2 lines, respectively. Whereas, 41% seeds of the vector control plants germinated under the same growth condition. On the other hand, 85% and 74% seeds of the TH1 and TH2 lines, respectively, germinated in the presence of 0.4 M mannitol compared to 51% seeds of the vector control. To further assess abiotic stress tolerance, 5 days old seedlings, germinated on MS media, were placed onto MS+ mannitol/NaCl media and further grown for 10 days. Compared to the vector control, TH1 and TH2 lines exhibited higher growth rate and accumulated more biomass (Fig. 9B,C). These results suggested a potential role of *ObTLP1* in mediating abiotic stress tolerance in transgenic Arabidopsis.

## Discussion

In the present study, a thaumatin-like protein ObTLP1 has been identified from an EST library that was previously prepared from the MeJA-elicited *O. basilicum*^31^. *ObTLP1* has been functionally characterized using bacterially-expressed recombinant protein and also following ectopic expression in Arabidopsis. ObTLP1 showed sequence similarity with the PR5 members of other plant species and all the sixteen conserved cysteine residues that are required for generating eight disulphide bonds in TLPs, were identified (Figs 2 and 3A). An N-terminal signal peptide was recognised in ObTLP1 (Fig. 3A), suggesting it to be a secretory protein like other TLPs that are localized to the extracellular space^9^,^11^. Phylogenetic analysis showed that ObTLP1 is closely related to the sweet-tasting protein, thaumatin of *T. danielli* (Fig. 2). Moreover, ObTLP1 showed high degree of structural and sequence identities (54.67%) with thaumatin (Fig. 3A–D). Besides, ObTLP1 also showed 50.88% sequence identity with the stress responsive protein osmotin of *N. tabacum*. Induced accumulation of *ObTLP1* transcript in *O. basilicum* after MeJA, SA, ABA, ACC treatments was noticed (Fig. 1A–D). Thus, *ObTLP1* might act downstream of multiple hormone signalling pathways that are involved in mediating biotic and abiotic stress responses in plants^2^,^26^,^27^,^28^. Moreover, induced expression of *ObTLP1* under biotic and abiotic stresses suggested a potential role of *ObTLP1* in mediating multiple stress responses in *O. basilicum* (Fig. 1F–H). Similarly, expression of TLPs of other plant species was also reported to be responsive to multiple stress conditions and hormonal treatments^9^,^18^,^41^. Under unstressed condition, the expression of *ObTLP1* appears to be organ-preferentially regulated in *O. basilicum* (Fig. 1E). Maximum transcript level was recorded in inflorescences, followed by leaves, stems and roots. Although under unstressed condition low transcript level was observed in roots, under abiotic stress conditions maximum transcriptional upregulation of *ObTLP1* was noticed in roots compared to leaves (Fig. 1G). Under dehydration, roots and leaves accumulated equivalent level of *ObTLP1* transcript (Fig. 1H). However, after 48 hr of salt treatment, *ObTLP1* transcript level was found to be more in roots than in leaves (Fig. 1H). Taken together, these results suggested that *ObTLP1* expression is organ-preferential under unstressed condition, and inducible under biotic and abiotic stress conditions.

Ectopic expression of *ObTLP1* in Arabidopsis conferred tolerance to necrotrophic fungal pathogens *S. sclerotiorum* and *B. cinerea* (Figs 6A–C and 7A–C). As *E. coli*-expressed recombinant ObTLP1 showed antifungal activity against these fungi (Fig. 4A,B), enhanced fungal tolerance of the *ObTLP1*-expressing Arabidopsis might be attributed to the hindrance of successful host colonization by these fungi in the presence of the antifungal ObTLP1 in cells. As suggested for other TLPs, antifungal activity of ObTLP1 might be attributed to its binding to the fungal cell wall components 1,3-β -glucan and phosphomannoproteins, and/or plasma membrane receptor-like proteins that finally lead to increased plasma membrane permeability and induction of apoptosis in fungi^20^,^21^,^42^,^43^,^44^. However, further studies need to be carried out to assign this mode of action for the ObTLP1. The transcript level of *ObTLP1* was upregulated when *O. basilicum* was challenged with *S. sclerotiorum* (Fig. 1F); suggesting a potential biological role of *ObTLP1* in defense against fungal pathogens. As *ObTLP1* expressed at the highest level in *O. basilicum* inflorescences, its biological function in protecting inflorescences from fungal infections cannot be excluded.

Analysis of the transcript expression patterns of the plant defense marker genes that are controlled through SA and JA/ET signalling pathways^40^,^45^, revealed increased transcript expression in *ObTLP1*-expressing Arabidopsis compared to the vector control, when plants were challenged with the fungus (Fig. 8B). The increased expression of *PDF1.2* and *VSP1* in *ObTLP1*-expressing Arabidopsis, compared to the vector control, suggested that JA/ET-mediated defense response was more active in cells in the presence of ObTLP1. The induction of the JA/ET pathway was quite expected as compared to the SA pathway because JA/ET pathway is the major mediator of defense response against necrotrophic pathogens like *S. sclerotiorum* and *B. cinerea*^25^. However, under unstressed conditions, the transcript levels of the defense marker genes in *ObTLP1*-expressing Arabidopsis and vector control plants remained unaltered (Fig. 8A). This suggested that under normal growth condition, ObTLP1 possibly did not play any role in modulating gene expression related to the plant defense response. Therefore, it is possible that some additional factor(s) which is(are) expressed in plants after fungal challenge determines the function of ObTLP1 in manipulating gene expression-related to plant defense response. However, this outcome on defense gene expression may be an indirect effect caused by slow spreading of the fungus in *ObTLP1*-expressing Arabidopsis because of the fungal growth inhibition by ObTLP1 (Fig. 4A,B). Thus, compromised host colonization by the fungus might have resulted in induced plant defense responses as marked by the defense-related gene expressions (Fig. 8B).

In addition to fungal pathogens, *ObTLP1*-expressing Arabidopsis were also tolerant to dehydration and salt stress conditions created by the applications of mannitol and NaCl, respectively (Fig. 9A–C). As compared to the vector control, *ObTLP1*-expressing Arabidopsis displayed better seed germination, and growth under dehydration and salt stress conditions. Both salt treatment and dehydration cause osmotic stress in cells. *N. tabacum* osmotin, an orthologue of ObTLP1 that was previously suggested to be involved in osmotic adjustment in cells by facilitating the accumulation or compartmentalization of solutes^24^ also improved tolerance to abiotic stresses^46^,^47^ in transgenic plants, in addition to providing tolerance to the fungal pathogens^47^,^48^. Thus, dehydration and salt stress tolerance of the *ObTLP1*-expressing Arabidopsis suggested a potential involvement of ObTLP1 in maintaining osmotic adjustment in cells during abiotic stress conditions. *ObTLP1* transcript level was upregulated when *O. basilicum* was exposed to dehydration and salt stress conditions; suggesting a possible role of the *ObTLP1* in mediating abiotic stress responses in *O. basilicum* as well (Fig. 1G,H).

In conclusion, this work led to the identification and functional characterization of an *O. basilicum* PR5 family member that is responsive to multiple stresses and hormonal elicitations. Taken together, the results highlight the role of ObTLP1 in mediating tolerance to the fungal pathogens and to the abiotic stresses by hindering fungal colonization in host and by maintaining osmotic adjustment in cells, respectively. Fungal pathogen and abiotic stress tolerance of the *ObTLP1*-expressing Arabidopsis, suggest that *ObTLP1* might be a useful candidate for providing tolerance in crops towards multiple stresses.

## Methods

### Plant materials and growth conditions

*O. basilicum* (CIM-Saumya) growth conditions were described previously^31^. *A. thaliana* (ecotypes *Col*-*0*) seeds were surface sterilized (2% sodium hypochlorite, 0.02% Triton X-100) and were spread onto MS agar plates and kept for 3 days at 4 °C. Plates were further transferred to the growth chamber (Conviron) with 16/8 hr light/dark cycle at 22–25 °C and 50–60% relative humidity. Fungal strains were routinely maintained on sterilized potato dextrose (PD) agar media at 22–25 °C temperature.

### Hormone treatments

*O. basilicum* plants were sprayed with different concentration of elicitors such as 250 μ M MeJA^31^, 100 μ M ACC (MilliQ water with 0.015% DMSO and 0.05% Triton X-100), and 500 μ M SA and 100 μ M ABA (in MilliQ water with 0.05% Triton X-100). For control, plants were sprayed with same solution without addition of elicitor and maintained under same growth condition as elicitor-treated plants. Samples were collected at regular intervals, quick-frozen in liquid nitrogen, and stored at − 80 °C until further use.

### Cloning and sequence analysis of *ObTLP1*

*ObTLP1* EST sequence (JZ190528) was obtained from the MeJA-elicited *O. basilicum*^31^ and the full-length cDNA sequence (JQ793640) was determined following 3′ RACE as per manufacturer’s instructions (Invitrogen). Phylogenic analysis^49^ and multiple sequence alignment (ClustalW2; http://www.ebi.ac.uk) of ObTLP1 with orthologs of other plant species, were carried out.

### Homology modeling

MODELLER 9.13^50^ was used in this study to generate an initial 3D structure model for the ObTLP1. Thaumatin (PDB ID: 2VHK) of *T. daniellii* was selected as a best template on the basis of its atomic resolution (0.94 Å). Secondary structure prediction was done by using PDBsum (Supplementary Fig. S2). Prosa-web (www.prosa.services.came.sbg.ac.at/prosa.php) and WHAT IF (http://swift.cmbi.ru.nl/whatif/) programs were used for protein structure analysis to calculate overall model quality and to check packing quality of the modeled structure, respectively^51^ (Supplementary Fig. S4B,C). The quality of the model was estimated by plotting dihedrals Φ and Ψ onto Ramachandran plot (Supplementary Fig. S4A). Superimposition of the modeled ObTLP1 over the template 2VHK was done in discovery studio (Accelrys Software Inc.) to determine structural conservation between generated 3D model and template. The Structure Analysis and Verification Server (SAVES) was used for checking the stereochemical quality of the 3D structure.

### Bacterial expression, purification, and antifungal assay

*ObTLP1* open reading frame (ORF) of 681bp was PCR-amplified from cDNA using Phusion high-fidelity DNA polymerase (Finnzymes) and inserted into pET-28a(+) vector by using BamHI and XhoI restriction enzymes. After verifying the integrity of the *ObTLP1* through sequencing, expression plasmid (pET-*ObTLP1*) was introduced into *E. coli* BL21-CodonPlus (DE3)-RIPL (Agilent) following the standard heat shock method. Bacterial cultures were grown in LB medium containing kanamycin (50 μ g/ml), and IPTG at a final concentration of 0.2 mM (Sigma) was added to the cultures when O.D of the cultures reached to 0.6. Cultures were further grown at 18^o^C for 16 hr in induction medium and collected by centrifugation at 5000 rpm for 10 min at 4 °C. Cells were resuspended in lysis buffer (50 mM NaH_2_PO_4_, 300 mM NaCl, 10 mM imidazole, pH 8; with 0.5 mg/ml lysozyme) and disrupted following sonication as describe previously^52^. Poly (His)-tagged protein was purified using Ni-NTA agarose beads according to the manufacturer’s protocol (Qiagen) as described previously^52^. The purity of the protein was determined by resolving on 10% (w/v) SDS-PAGE gel. Antifungal activity of the purified protein towards phytopathogenic fungi *S. sclerotiorum* and *B. cinerea* was performed following agar disc diffusion method^9^. Fungal strains were initially cultured on PD with 1.5% agar (Himedia) for 1–2 days at 22–23 °C temperature. After that, five sterile Whatman filter paper (No. 42) discs of 0.9 cm diameter were placed onto plates at equal distance from the advancing edge of the growing fungal mycelia. Different concentrations of ObTLP1 purified protein was added to the Whatman filter paper discs. Only buffer (50 mM NaH_2_PO_4_, 300 mM NaCl, pH 8) and BSA in buffer were added to the discs to serve as the negative controls. Plates were incubated at 22–23 °C for 24 hr. For each of two independent experiments, fungal strains were tested in three separate plates.

### Development of transgenic Arabidopsis

For CaMV35S promoter-driven expression of *ObTLP1* in Arabidopsis, ORF was amplified from cDNA using Phusion high-fidelity DNA polymerase and inserted into pBI121 expression vector using XbaI and Ecl136II restriction enzymes. After verifying the integrity of the *ObTLP1* through sequencing, expression plasmid (pBI-*ObTLP1*) was introduced into *Agrobacterium tumefaciens* strain EHA105 following the freeze-thaw transformation method^53^. *Agrobacterium*-mediated transformation of Arabidopsis was carried out by following the floral dip method^54^. Positive transformants were selected on MS medium with 100 μ g/ml kanamycin (Sigma), and transplanted into SoilriteTM Mix (Keltech Energies Ltd, Bangalore) for growth until maturation. For isolating homozygous transgenic lines, seeds were germinated for successive generations on MS medium with 100 μ g/ml kanamycin.

### Genomic DNA isolation, PCR and qPCR

Genomic DNA was isolated following CTAB method^55^ and quantified using a nanodrop (Thermo Scientific). Genomic integration of the *ObTLP1* was confirmed following PCR using gene-specific primers and genomic DNA as template. In order to quantify fungal biomass in infected leaves, genomic DNA isolated from infected leaves was used as template for qPCR analysis using SYBR *Premix Ex Taq* (Tli RNase H Plus) and ROX Reference Dye II (Takara). Relative level of *S. sclerotiorum* ITS genomic DNA (KC748491) or *B. cinerea Actin* genomic DNA (NW_001814525) was determined using Arabidopsis chloroplast-encoded ribulose-1,5-bis-phosphate carboxylase/oxygenase large subunit (Atcg00490) as reference gene, as described previously^39^. Primer sequences are provided in the Supplementary Table S2.

### RNA Isolation, sqRT-PCR and qRT-PCR

Total RNA was isolated using RNAiso Plus according to the manufacturer’s instructions (Takara). Unless otherwise stated, three independent isolations with four plants for each group were performed. RNA was quantified using a nanodrop (Thermo Scientific), and treated with DNaseI according to the manufacturer’s instructions (Thermo Scientific). For cDNA synthesis, RNA (2 μ g) was reverse transcribed using MultiScribe reverse transcriptase according to the manufacturer’s instructions (Applied Biosystems) in 20 μ l total reaction volume and diluted three times with MilliQ water. In order to confirm *ObTLP1* expression in transgenic lines, sqRT-PCR was carried out by using *Taq* DNA polymerase (Invitrogen) in 25 μ l reaction volume for 28 PCR cycles in a Thermal Cycler (Agilent technologies). qRT-PCR was performed using 7900 HT Fast Real-Time PCR (Applied Biosystems) in 10 μ l reaction volume consisting of 5 μ l SYBR *Premix Ex Taq* (Tli RNase H Plus), 0.2 μ l ROX Reference Dye II (Takara), 0.5 μ l of diluted cDNA, and 500 nM of each forward and reverse gene-specific primers. Relative transcript expression level was calculated using the 2^−ΔΔct^ method as described previously^56^ using *ObACT* (*O. basilicum*) or *AtUBC21* (Arabidopsis) as endogenous control. For choosing a suitable reference gene as endogenous control in qRT-PCR, traditional reference genes for *O. basilicum* (*ObACT*, *ObEF-1α, ObGAPDH, ObTIP41, ObTUB, ObUBC21, ObUBQ5, ObUBQ10*) and Arabidopsis (*AtACT2, AtEF-1α, AtGAPDH, AtTUB4, AtUBC21, AtUBQ10*) were evaluated with geNorm^57^ and NormFinder^58^ software. For *O. basilicum*, *ObACT* showed most stable expression under the present experimental conditions (Supplementary Fig. S5A–C). For Arabidopsis, *AtUBC21* was among the stably expressed genes with geNorm M value below the critical value of 0.5 (Supplementary Fig. S6A–D). *AtUBC21* was also recognised as a stably expressed gene in previous study^59^. Primer sequences are shown in Supplementary Table S2.

### Biotic and abiotic stress treatments

Plants were infected with *S. sclerotiorum* mycelial suspension or *B. cinerea* spore suspension as described previously^39^, with few modifications. *S. sclerotiorum* and *B. cinerea* were initially cultured on potato dextrose (PD) agar plates. The advancing edge of growing *S. sclerotiorum* mycelia was subcultured into ½ PD broth and kept under continuous shaking (175 rpm) at 22–23 °C temperature for 2 days. Fresh mycelia (2 gm) of *S. sclerotiorum* was taken and homogenized into ½ PD broth with the help of a mortar-pestle. Mycelial suspension of *S. sclerotiorum* (2 gm wet mycelia in 5 ml ½ PD broth) was prepared. *B. cinerea* spores were harvested with ¼ PD broth from one-week-old culture by gentle rubbing of the fungal surface, and filtered through muslin cloth to remove mycelia. Adaxial surface of four rosette leaves of four-week-old Arabidopsis plants were inoculated with 5 μ l *S. sclerotiorum* mycelial suspension or *B. cinerea* spore suspension (10^5^ spores/ml). For *O. basilicum*, 20 μ l mycelial suspension of *S. sclerotiorum* was used for leaf infection. Inoculated plants were kept in a growth tray with a transparent cover to maintain high humidity, and a 16 hr photoperiod at 23 °C in a growth chamber was followed. For phenotypic characterization, lesion diameters of the infected leaves were measured with a scale at different time intervals before disease symptoms expanded beyond the inoculated leaves. For gene expression analysis, leaves from infected and uninfected plants were collected at different time points, quickly frozen with liquid N_2_ and stored at − 80 °C until further use. For abiotic stress treatments, surface sterilized seeds of transgenics were germinated on MS plates containing 100 μ g/ml kanamycin. Plates were placed at 4 °C in dark for three days and further transferred to tissue culture, and maintained at 23–25 ^o^C with 16/8 h light/dark cycle. Seedlings were transferred to new MS plates containing NaCl (75 mM and 150 mM) for salt stress treatment and mannitol (0.3 M and 0.4 M) for dehydration, and further grown for additional 10 days. Plants were grown at 23–25 °C under a 16/8-h light/dark cycle. In order to determine seed germination rates of the vector control and *ObTLP1*-expressing Arabidopsis under abiotic stress, seeds were germinated for eight days on MS plates containing NaCl (75 mM and 150 mM) or mannitol (0.3 M and 0.4 M). For salt stress treatment to *O. basilicum*, four-to-five leaves stage plants were watered with 250 mM NaCl, and for dehydration watering was withheld. Plants watered regularly served as control for the dehydration and salt stress.

## Additional Information

**Accession codes**: The complete cDNA sequence of *ObTLP1* was submitted to the GenBank database under the accession number JQ793640.

**How to cite this article**: Misra, R. C. *et al.* A thaumatin-like protein of *Ocimum basilicum* confers tolerance to fungal pathogen and abiotic stress in transgenic Arabidopsis. *Sci. Rep.* **6**, 25340; doi: 10.1038/srep25340 (2016).

## Supplementary Material

Supplementary Information
