# Supplementary material for: A thaumatin-like protein of Ocimum basilicum confers tolerance to fungal pathogen and abiotic stress in transgenic Arabidopsis
Source: Sci Rep. 2016 May 6;6:25340. doi: 10.1038/srep25340 (PMC4858651; doi:10.1038/srep25340)
Supplement: Supplementary Information [file srep25340-s1.pdf]

Manuscript Title:

A thaumatin-like protein of *Ocimum basilicum* confers tolerance to fungal pathogen and abiotic stress in transgenic Arabidopsis

Author List:

Rajesh Chandra Misra, Sandeep, Mohan Kamthan, Santosh Kumar and Sumit Ghosh*****

Supplementary Information

Supplementary Figure S1-S7

Supplementary Table S1-S2

```
Ocimumbasilicum_AGX15390.1_         MISLKFIST----LLPF-LLLLHA-ATAIRFDIQNKCSYTIWPAVLP--- 41
ObTLP1_JQ793640_                    MNSLKFIST----LLPFLLLLLHA-ATAIRFDIQNKCSYTIWPAVLP--- 42
Thaumatococcusdaniellii_P02884      MAATTCFFF----LFPF-LLLLTL-SRAATFEIVNRCSYTVWAAASKGD- 43
Solanumnigrum_AGH14263.1_           MGYL-RSSF-----VFFLLAFVTY-TYAATIEVRNNCPYTVWAASTPI-- 41
Solanumnigrum_AAL87640.1_           MGYS-RSSF-----VFFLLTFVTY-TYATSFEVRNNCPYTVWAASTPI-- 41
Nicotianatabacum_P14170.2_          MGNL-RSSF-----VFFLLALVTY-TYAATIEVRNNCPYTVWAASTPI-- 41
Actinidiachinensis_AGC39177.1_      MGYL-TMAFG--LPVFLLLTLFSS-AHAATFEIRNNCPYTVWAAASP--- 43
Ficuspumilavar.Awkeotsang_ABB8      MGSLANLSII--FSLIFLTLFFSS-SNAASFEIRNECSYPVWAAASP--- 44
Camelliasinensis_ABE01396.1_        MSFPKSLTIL--FPLLF-TLLIPF-TNAATFAVLNKCTYTVWAAASP--- 43
Arabidopsisthaliana_CAA61411.1      MANLLVSTF-----IFSALLLIST-ATAATFEILNQCSYTVWAAASP--- 41
Glycinemax_P25096.1_                ----------------------------ARFEITNRCTYTVWAASVPV-- 20
Vitisvinifera_AAQ10092.1_           MGLCKILSI---SSFLLTALFFTP-SYAATFNIQNHCSYTVWAAAVP--- 43
Actinidiachinensis_AGC39176.1_      MSTFKSLSL---SALLFIAFLFTC-ARGATFNIINNCPFTVWAAAVP--- 43
Fragariaxananassa_AAF13707.1_       MSLLKNLP----TVLSILYFAAST-VNAATFNKKNNCPFTVWAGAVP--- 42
Sambucusnigra_AAK59275.1_           MGFLKSLPI---SIFFVIALISSS-AYAANFNIRNNCPFTVWAAAVP--- 43
Vincetoxicummongolicum_ADO3289      MN-FHHFSS---LLVLVLSCLFAT-SYAATFVVTNNCQYPVWAAGVPV-- 43
Oryzasativa_P31110.1_               MASPATSSAV--LVVVLVATLAAGGANAATFTITNRCSFTVWPAATP--- 45
Pyruspyrifolia_AEO36939.1_          MSMMKNQVA--SLLGLTLAILFFSGAHAAKITFTNNCPYTVWPGTLTGDQ 48
Arachisdiogoi_ACT11052.1_           MAITR------VVLSLSFA-FFLCVAHGAQITLTNKCSYTVWPGSQANAN 43
Medicagotruncatula_AFK44890.1_      MASSSSSSTNSLLISTFIFLTIFSPSQALIFTFVNNCPYTVWPAIIPNGG 50
                                                                  :   *.* :.:*..

Ocimumbasilicum_AGX15390.1_         ----HGGGRRLDSGQTWTLSFQNGPKLAKVWARTNCTFDSSGKGKCLTGD 87
ObTLP1_JQ793640_                    ----HGGGRRLDSGQTWTLSFQNGPKLAKVWARTNCTFDSSGKGKCLTGD 88
Thaumatococcusdaniellii_P02884      -AALDAGGRQLNSGESWTINVEPGTKGGKIWARTDCYFDDSGRGICRTGD 92
Solanumnigrum_AGH14263.1_           -----GGGRRLDRGQTWVINAPRGTSMARIWGRTNCNFDGAGRGSCQTGD 86
Solanumnigrum_AAL87640.1_           -----GGGRRLDRGQTWVINAPRGTSMARIWGRTNCNFDGAGRGSCQTGD 86
Nicotianatabacum_P14170.2_          -----GGGRRLDRGQTWVINAPRGTKMARVWGRTNCNFNAAGRGTCQTGD 86
Actinidiachinensis_AGC39177.1_      -----GGGRRLNSKQQWNLNVAAGTKMARIWGRTKCNFDGSGRGHCETGD 88
Ficuspumilavar.Awkeotsang_ABB8      -----GGGRRLDPTQNWILNVPAGTSMARIWGRTNCNFDGAGRGRCQTGD 89
Camelliasinensis_ABE01396.1_        -----GGGMRLDPGQSWTVNVNPGTTQARIWGRTNCNFDANGNGQCQTGD 88
Arabidopsisthaliana_CAA61411.1      -----GGGRRLDAGQSWRLDVAAGTKMARIWGRTNCNFDSSGRGRCQTGD 86
Glycinemax_P25096.1_                -----GGGVQLNPGQSWSVDVPAGTKGARVWARTGCNFDGSGRGGCQTGD 65
Vitisvinifera_AAQ10092.1_           -----GGGMQLGSGQSWSLNVNAGTTGGRVWARTNCNFDASGNGKCETGD 88
Actinidiachinensis_AGC39176.1_      -----GGGKRLDRGQNWIINPGAGTKGARVWARTGCNFDGAGRGKCQTGD 88
Fragariaxananassa_AAF13707.1_       -----GGGKQLGTGQTWTINVAAGTKGARIWPRTNCNFDGAGRGRCQTGD 87
Sambucusnigra_AAK59275.1_           -----GGGRQLNRGQVWSLDVPAGTRGARIWARTNCNFDGAGRGRCQTGD 88
Vincetoxicummongolicum_ADO3289      -----GGGRRLDRGQVWRLEVPAGTKQARIWGRTNCNFDASGRGKCETGD 88
Oryzasativa_P31110.1_               ----VGGGVQLSPGQTWTINVPAGTSSGRVWGRTGCSFDGSGRGSCATGD 91
Pyruspyrifolia_AEO36939.1_          KPQLSLTGFELASKASQSVDAP-FPWSGRFWGRTRCSTNAAGKFTCETAD 97
Arachisdiogoi_ACT11052.1_           SAQLSTTGFELPTGQSKTVDVP-APWSGKFWARTGCS-NNNGVFSCATAD 91
Medicagotruncatula_AFK44890.1_      FPVLSSSGFELRHFTHLSIPVPDTHWAGRAWARTGCS-TANNKFSCLTGD 99
                                           * .*       :        .: * ** *     .   * *.*
```

Supplementary Figure S1. Amino acid sequence comparison of *O. basilicum* ObTLP1 with PR5 members of other plant species. Multiple sequence alignment was carried out by using ClustalW2 program (http://www.ebi.ac.uk/Tools/msa/clustalw2/). Identical amino acid residues are depicted as *, while conserved and semi-conserved substitutions are marked as : and ., respectively. Sixteen cysteine resides that are conserved in TLPs are highlighted.

```
Ocimumbasilicum_AGX15390.1_         CGG-QLNCTTFG-SPPHTKAEYGLNDFGRKDY--YDVSVLDGYNLPMEMT 133
ObTLP1 JQ793640                     CGG-QLNCTTFG-SPPHTKAEYGLNDFGRKDY--YDVSVLDGYNLPMEMT 134
Solanumnigrum_AAL87640.1_           CGG-VLQCTGWG-KPPNTLAEYALNQFSNLDF--WDISLVDGFNIPMTFA 132
Nicotianatabacum_P14170.2_          CGG-VLQCTGWG-KPPNTLAEYALDQFSGLDF--WDISLVDGFNIPMTFA 132
Actinidiachinensis_AGC39177.1_      CGG-VLECKGWG-VPPNTLAEYALNQFGNLDF--FDISLVDGFNIPMDFS 134
Ficuspumilavar.Awkeotsang_ABB8      CGG-LLQCQGWG-NPPNTLAEHALNQFGNLDF--YDISLVDGFNIPMSFG 135
Camelliasinensis_ABE01396.1_        CNG-LLQCQGYG-KAPNTLAEFALNQPNNLDF--VDISNVDGFNIPMDFS 134
Arabidopsisthaliana_CAA61411.1      CSG-GLQCTGWG-QPPNTLAEYALNQFNNLDF--YDISLVDGFNIPMEFS 132
Glycinemax_P25096.1_                CGG-VLDCKAYG-APPNTLAEYGLNGFNNLDF--FDISLVDGFNVPMDFS 111
Vitisvinifera_AAQ10092.1_           CGG-LLQCTAYG-TPPNTLAEFALNQFSNLDF--FDISLVDGFNVPMAFN 134
Actinidiachinensis_AGC39176.1_      CNG-LLQCQAFG-QPPNTLAEYALNQFNNLDF--FDISLVDGFNVAMEFS 134
Fragariaxananassa_AAF13707.1_       CGG-LLQCQGYG-QPPNTLAEYALNQYMNRDF--YDISLIDGFNVPMDFS 133
Sambucusnigra_AAK59275.1_           CNG-LLSCQAYG-APPNTLAEYALNQFNNLDF--FDISLVDGFNVAMDFS 134
Vincetoxicummongolicum_ADO3289      CNG-LLQCKNFG-SPPNTLAEFALNQFANKDF--FDISLVDGFNVPMDFS 134
Oryzasativa_P31110.1_               CAG-ALSCTLSG-QKPLTLAEFTIG--GSQDF--YDLSVIDGYNVAMSFS 135
Pyruspyrifolia_AEO36939.1_          CGSGQVACNGAGAIPPATLVEITIAANGGQDF--YDVSLVDGFNLPMSVA 145
Arachisdiogoi_ACT11052.1_           CGN-HLECSGAGEATPASLMEFTIASNGGQDF--YDVSNVDGFNVPSSIT 138
Medicagotruncatula_AFK44890.1_      CGN-SLQCHGAGGSPPATLVQFDVH-HGNKDFSSYSVSLVDGFNTPLTVT 147
                                    * .  : *   *   * :  :  :      *:   .:* :.*:* .  .

Ocimumbasilicum_AGX15390.1_         PTAN--GCTRSVKCAAEDIVANCPSQLKVDGG---------CQNPCTVFK 172
ObTLP1_JQ793640_                    PTTN--GCTRSVKCAAEDIVANCPSQLKVDGG---------CQNPCTVFK 173
Thaumatococcusdaniellii_P02884      PTTR--GC-RGVRCAAD-IVGQCPAKLKAPGG--------GCNDACTVFQ 175
Solanumnigrum_AGH14263.1_           PTNPSGGKCHSIQCTAN-INGECPAALRVPGG---------CNNPCTTFG 172
Solanumnigrum_AAL87640.1_           PTNPSGGKCHSIQCTAN-INGECPAALRVPGG---------CNNPCTTFG 172
Nicotianatabacum_P14170.2_          PTNPSGGKCHAIHCTAN-INGECPRELRVPGG---------CNNPCTTFG 172
Actinidiachinensis_AGC39177.1_      PTRSPSGKCRPISCTAN-IIGQCPNPLKTAGG---------CHNPCTVFK 174
Ficuspumilavar.Awkeotsang_ABB8      PTR---GGCKVIGCTAD-INGQCPNELRTPGG---------CQNPCTVFK 172
Camelliasinensis_ABE01396.1_        PTT---AVCKSLRCAAN-IVGECPAELQTPGG---------CNNPCTVYK 171
Arabidopsisthaliana_CAA61411.1      PTS---SNCHRILCTAD-INGQCPNVLRAPGG---------CNNPCTVFQ 169
Glycinemax_P25096.1_                PTSN--GCTRGISCTAD-INGQCPSELKTQGG---------CNNPCTVFK 149
Vitisvinifera_AAQ10092.1_           PTSN--GCTRGISCTAD-IVGECPAALKTTGG---------CNNPCTVFK 172
Actinidiachinensis_AGC39176.1_      PTSG--GCTRGIKCTAN-INEQCPNELRAPGG---------CNNPCTVFK 172
Fragariaxananassa_AAF13707.1_       PVSN--GCTRGIRCTAD-INGQCPAQLRAPGG---------CNNACTVSK 171
Sambucusnigra_AAK59275.1_           PTG---GCARGIQCTAD-INGQCPNELRAPGG---------CNNPCTVYR 171
Vincetoxicummongolicum_ADO3289      PTSN--GCSRGIKCAAQ-INRECPNQLKAPGG---------CNNPCTVFK 172
Oryzasativa_P31110.1_               CSSG-----VTVTCRDS--------------------------------- 147
Pyruspyrifolia_AEO36939.1_          PQGGT-GDCKPSSCPAN-VNAACPAQLQVKA--ADGTVIA-CKSACLAFG 190
Arachisdiogoi_ACT11052.1_           PQGGS-GACNVASCPAN-INAACPAALQFKG--SDGSVIG-CKSACVEFG 183
Medicagotruncatula_AFK44890.1_      PHEGK-GECPVVGCKAD-LVASCPPVLQHRVPMGHGPVVA-CKSGCEAFH 194
                                                 *  .
```

Supplementary Figure S1. Continued.

```
Ocimumbasilicum_AGX15390.1_           TTEYCCHAGK-----CRPTDMSRFFKSRCRDAFTYPQDDPTST--FTCPE 215
ObTLP1_JQ793640_                      TTEYCCHAGE-----CRPTDMSRFFKSRCRDAFTYPQDDPTST--FTCPE 216
Thaumatococcusdaniellii_P02884        TSEYCCTTGK-----CGPTEYSRFFKRLCPDAFSYVLDKPT-T--VTCPG 217
Solanumnigrum_AGH14263.1_             GQQYCCTQGP-----CGPTELSRFFKQRCPDAYSYPQDDPTST--FTCPS 215
Solanumnigrum_AAL87640.1_             GQQYCCTQGP-----CGPTELSKFFKQRCPDAYSYPQDDPTST--FTCPS 215
Nicotianatabacum_P14170.2_            GQQYCCTQGP-----CGPTFFSKFFKQRCPDAYSYPQDDPTST--FTCPG 215
Actinidiachinensis_AGC39177.1_        TQEYCCTQGP-----CGPTNYSRFFKERCRDAYSYPQDDPTST--FTCPG 217
Ficuspumilavar.Awkeotsang_ABB8        TNEFCCTNGQG---SCGPTNFSKFFKDRCRDAYSYPQDDPTST--FTCPA 217
Glycinemax_P25096.1_                  TDQYCCNSGS-----CGPTDYSRFFKQRCPDAYSYPKDDPPST--FTCNG 192
Vitisvinifera_AAQ10092.1_             TDEYCCNSGS-----CNATDYSRFFKTRCPDAYSYPKDDQTST--FTCPA 215
Actinidiachinensis_AGC39176.1_        TDQYCCNSGN-----CGPTDYSRFFKTRCPDAYSYPKDDQTST--FTCPG 215
Fragariaxananassa_AAF13707.1_         TDQYCCNSGH-----CGPTDYSRFFKSRCPDAYSYPKDDATSTVLFTCPG 216
Sambucusnigra_AAK59275.1_             TNEYCCTNGQG---TCGPTNFSRFFKERCRDAYSYPQDDPTST--FTCPG 216
Vincetoxicummongolicum_ADO3289        TDQYCCNSGR-----CSPTNFSSFFKKRCPDAYSYPKDDQTST--FTCPA 215
Oryzasativa_P31110.1_                 ----------------------------RCPDAYLFPEDNTKTH---ACSG 167
Pyruspyrifolia_AEO36939.1_            DSKYCCTPPNNTPETCPPTEYSQFFEQQCPQAYSYAYDDKNST--FTCSG 238
Arachisdiogoi_ACT11052.1_             TPEYCCTGDHNTAATCPATNYSEFFSNQCPNAYSYAYDDKRGT--FTCSG 231
Medicagotruncatula_AFK44890.1_        SDEHCCRNHFNNPQTCKPTVYSKFFKDACPATFTFAHDSPSLI--HQCSS 242
                                                                  *  :: :  *.        *

Ocimumbasilicum_AGX15390.1_           ST-SYRVVFCP------------------------- 225
ObTLP1_JQ793640_                      ST-SYRVVFCP------------------------- 226
Thaumatococcusdaniellii_P02884        SS-NYRVTFCPTALELEDE----------------- 235
Solanumnigrum_AGH14263.1_             GSTNYRVVFCPNGVT----SPNLPLERPASTDKVAN 247
Solanumnigrum_AAL87640.1_             DSTNYRVVFCPNGVT----SPNFPLEMPSSTDEVAK 247
Nicotianatabacum_P14170.2_            GSTNYRVIFCPNGQA----HPNFPLEMPGS-DEVAK 246
Actinidiachinensis_AGC39177.1_        GS-NYRVTFCPNG------SPHFPLEMYGESDVE-- 244
Ficuspumilavar.Awkeotsang_ABB8        GA-NYKVVFCPRA------SAHFPLEMVKSASES-- 244
Camelliasinensis_ABE01396.1_          GT-NYAITFCP------------------------- 226
Arabidopsisthaliana_CAA61411.1        TN--YRVVFCPRSRLGATGSHQLPIKMVTEEN---- 244
Glycinemax_P25096.1_                  GT-DYRVVFCP------------------------- 202
Vitisvinifera_AAQ10092.1_             GT-NYEVVFCP------------------------- 225
Actinidiachinensis_AGC39176.1_        GT-NYKVVFCP------------------------- 225
Fragariaxananassa_AAF13707.1_         GT-NYRVVFCP------------------------- 226
Sambucusnigra_AAK59275.1_             GT-NYRVVFCP------------------------- 226
Vincetoxicummongolicum_ADO3289        GT-NYRVVFCP------------------------- 225
Oryzasativa_P31110.1_                 NS-NYQVVFCP------------------------- 177
Pyruspyrifolia_AEO36939.1_            GP-DYVITFCP------------------------- 248
Arachisdiogoi_ACT11052.1_             SP-NYAINFCP------------------------- 241
Medicagotruncatula_AFK44890.1_        PG-ELKVIFCH------------------------- 252
                                            : **
```

Supplementary Figure S1. Continued.

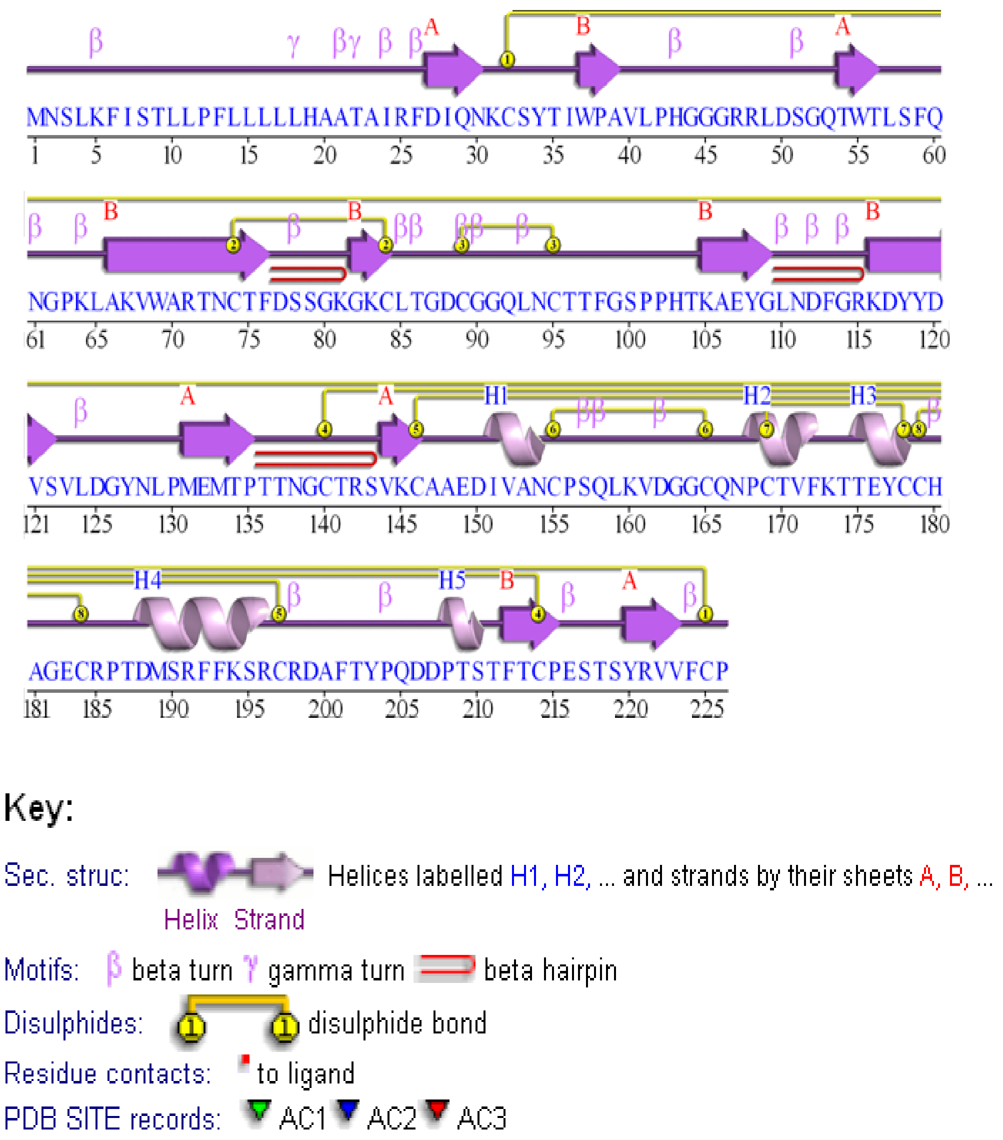

Supplementary Figure S2. A schematic presentation of the secondary structural elements in ObTLP1. α-helices are labeled with the letter 'H', and β-strands are lettered in uppercase. β, γ, and hairpin turns are also labeled. Eight disulphide bonds are mentioned with numbers 1 to 8. The secondary structure of *O. basilicum* ObTLP1 was determined using the PDBsum tool.

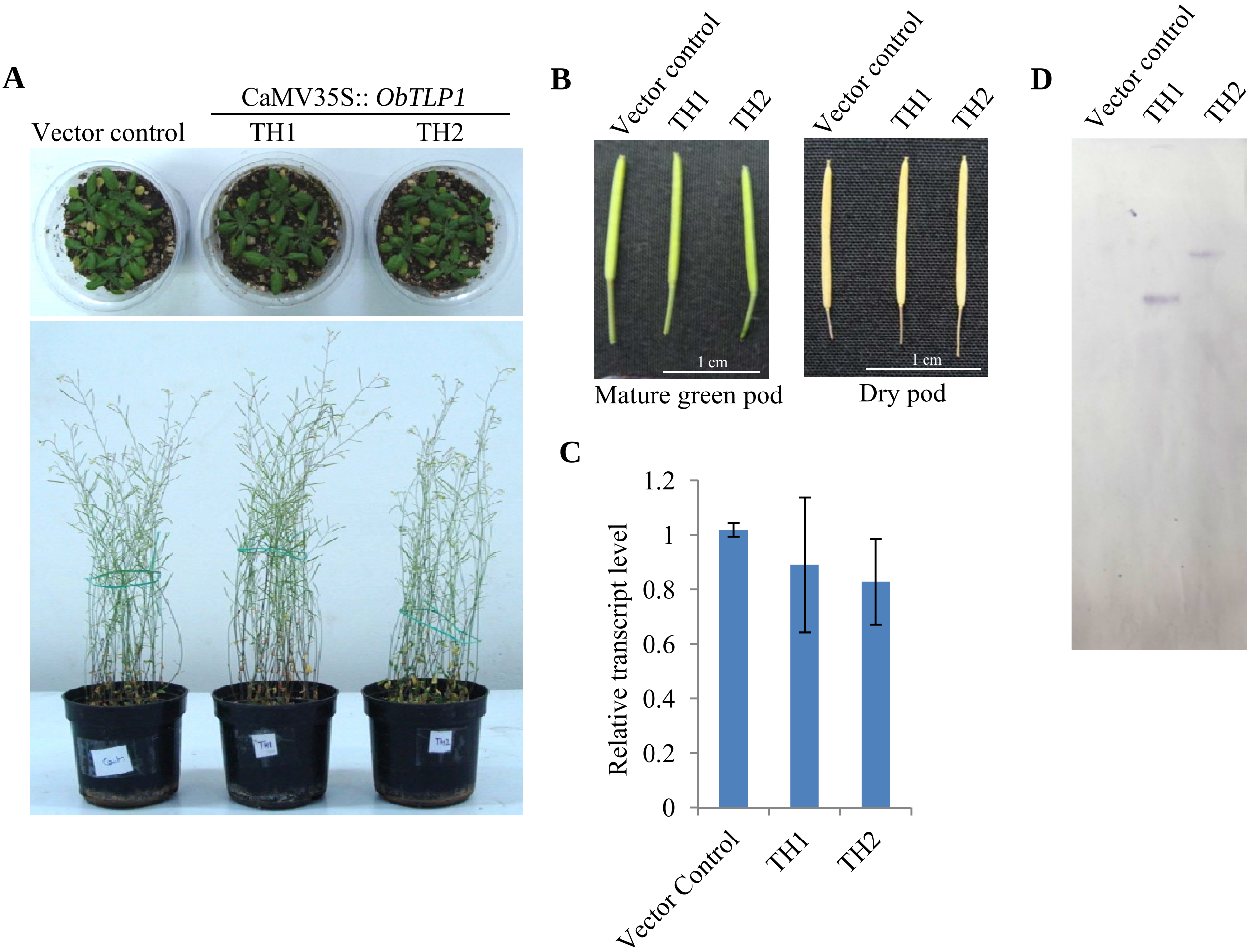

Supplementary Figure S3. Phenotypic and molecular characterization of *ObTLP1*-expressing Arabidopsis transgenic lines. (A) Photographs of vector control and *ObTLP1*-expressing Arabidopsis transgenic lines, taken at different growth stages. (B) Photographs of mature green and dry pods are shown. (C) Relative transcript level of Arabidopsis *AtOSM34* (AT4G11650), an ortholog of *ObTLP1,* was determined by qRT-PCR analysis. Data are mean ± s.d. from three biological replicates. (D) Southern blotting to determine transgene copy number in *ObTLP1*-expressing Arabidopsis transgenic lines. Genomic DNA (15 µg) was digested with XbaI and BamHI, precipitated with Ethanol/Sodium Acetate and electrophoresed on 0.8% Agarose gel. After depurination and denaturation, resolved DNAs were transferred onto Hybond N+ (GE Healthcare) following capillary method, and UV cross-linked. *ObTLP1* cDNA (725 bp) was DIG labelled and hybridized to membrane (DIG-System; Roche Diagnostics). Prehybridisation and hybridisation were performed at 65°C with standard buffer (5X SSC, 0.1% N-lauroylsarcosine, 0.02%SDS, 1% blocking reagent-Roche-11096176001). Detection was performed using an alkaline phosphatase-conjugated anti-digoxigenin antibody at 1:5000 dilutions (Roche-11093274910). After washing, blot was developed following colorimetric detection method using NBT and BCIP in detection buffer (0.1M Tris-HCl, pH-9.5 and 0.1M NaCl).

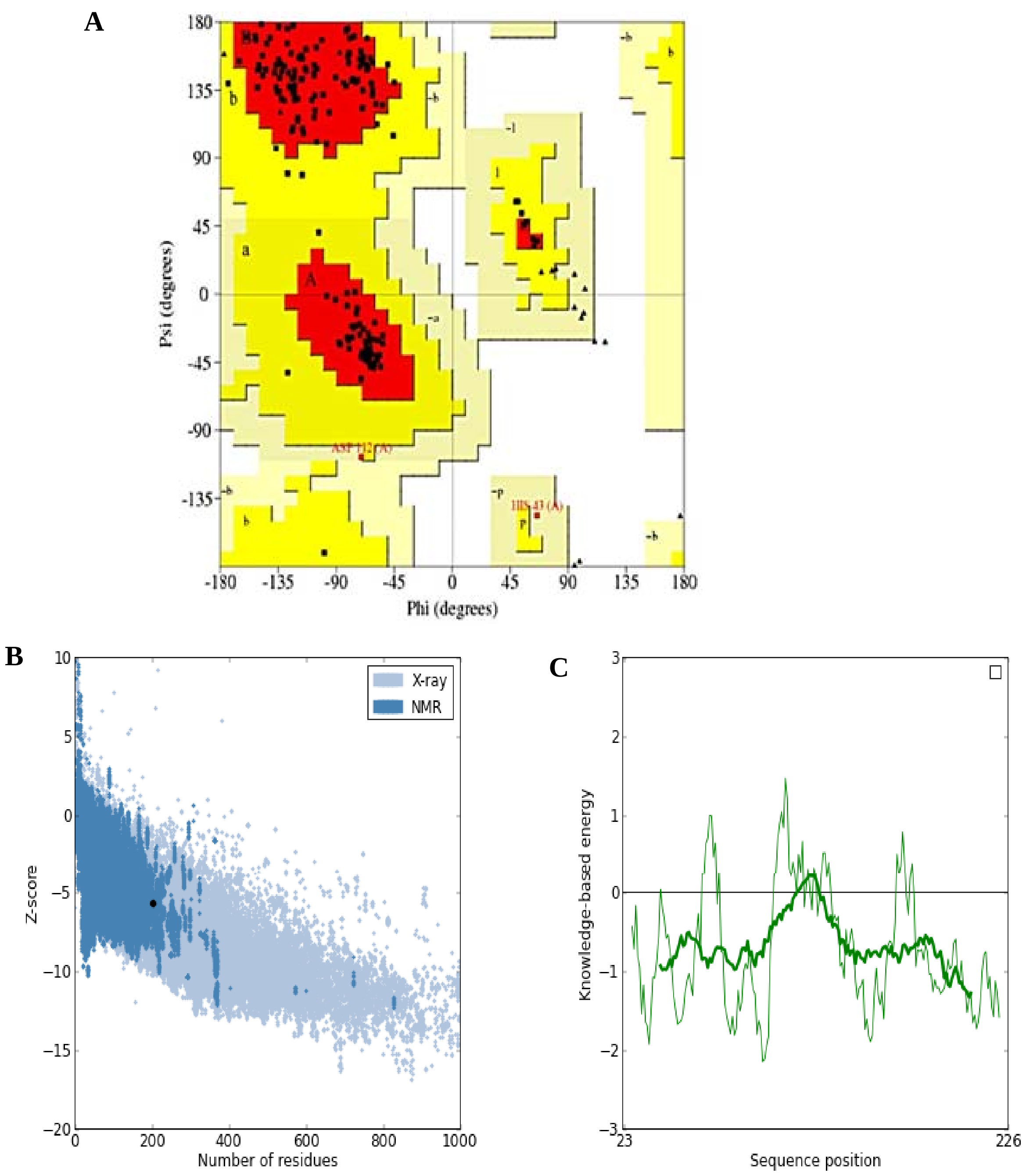

Supplementary Figure S4. Validation of the 3D structural model of ObTLP1. **(A)** Ramachandran plot was generated using PROCHECK program of Structure Analysis and Verification Server (SAVES) for the modeled ObTLP1. 94.1% of residues were found in the most favoured regions, 5.4% of residues were in the most allowed regions, and 0.5% of residues were found in the outlier regions. ProSA results showed that 3D model of ObTLP1 is very close to native structure (2VHK) determined by X-ray crystallography. **(B)** Z-score plot showing the quality of the predicted model in NMR region (dark blue). **(C)** Energy plot showing all residues of predicted model at very stable position (dark green).

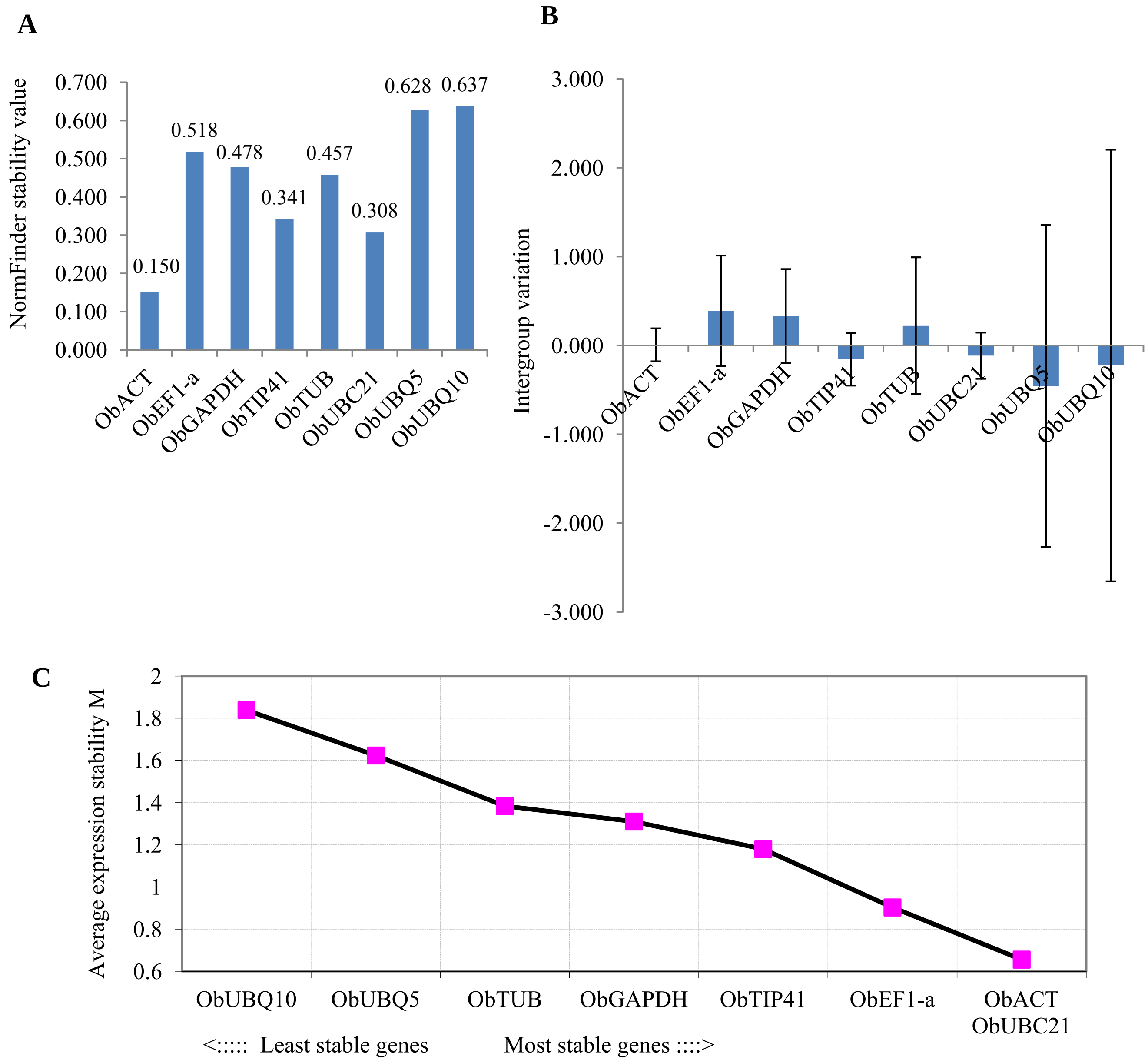

Supplementary Figure S5. Identification of stably expressed reference genes, following NormFinder and geNorm programs, for qRT-PCR analysis in *O. basilicum*. (A) Most stably expressed gene has lowest NormFinder stability value. (B) Inter-group variances for the reference genes obtained from NormFinder analysis. Error bars present the average of intra-group variance. An ideal reference gene has close to zero inter-group variation and low intra-group variation. cDNAs were prepared from total of sixteen samples comprising of two groups (normal and treated tissues) with eight samples each. (C) Expression stability (M) of the reference genes determined following geNorm program. Low M value means most stable reference gene.

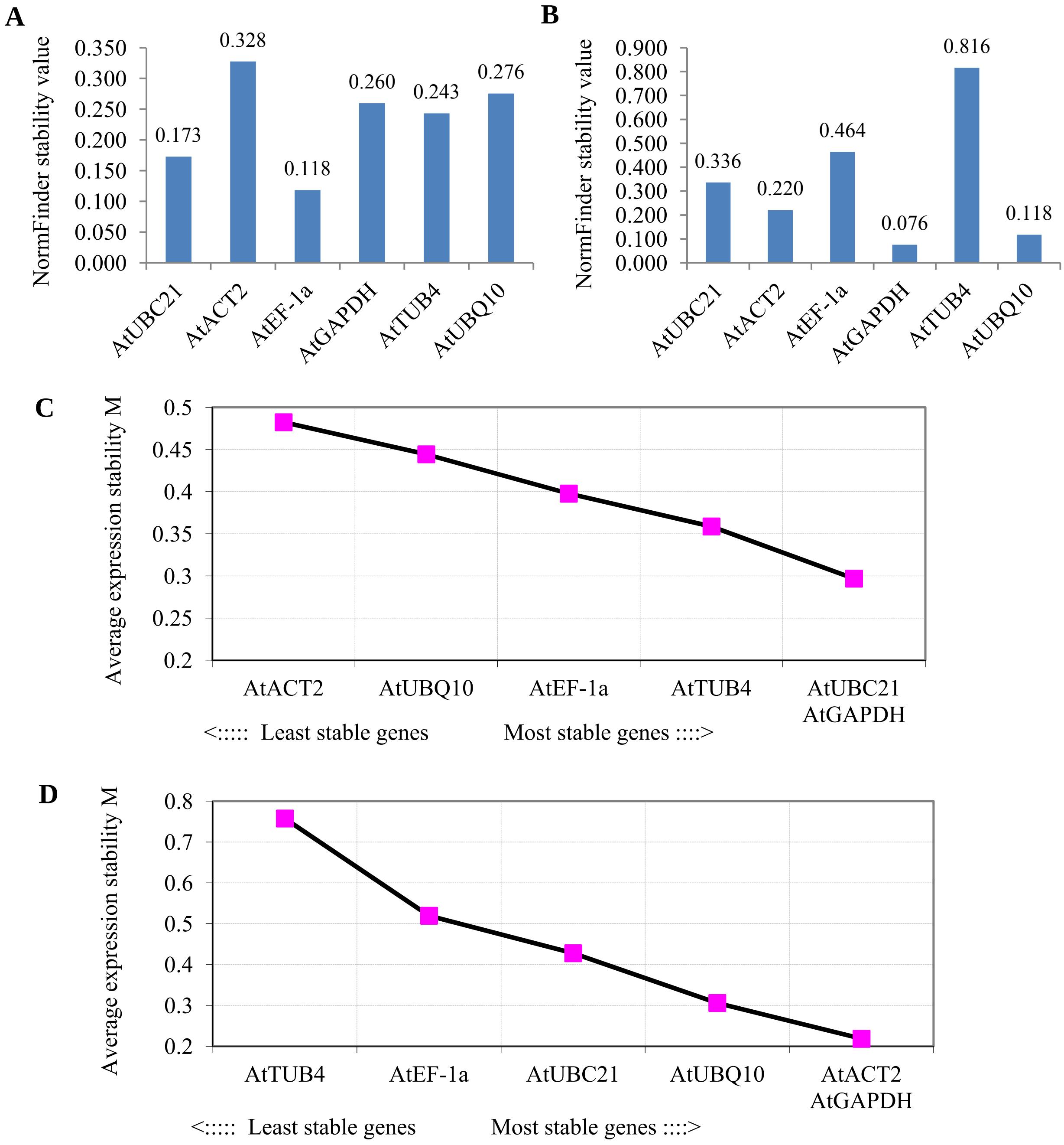

Supplementary Figure S6. Identification of stably expressed reference genes of Arabidopsis for qRT-PCR analysis. (A-B) NormFinder stability values of the reference genes for normal (A) and *S. sclerotiorum*-infected (B) leaf tissues. (C-D) Expression stability (M) of the reference genes determined following geNorm program for normal (C) and *S. sclerotiorum*-infected (D) leaf tissues. Twelve and six cDNA samples were used for normal (A, C) and *S. sclerotiorum*-infected (B, D) tissues, respectively.

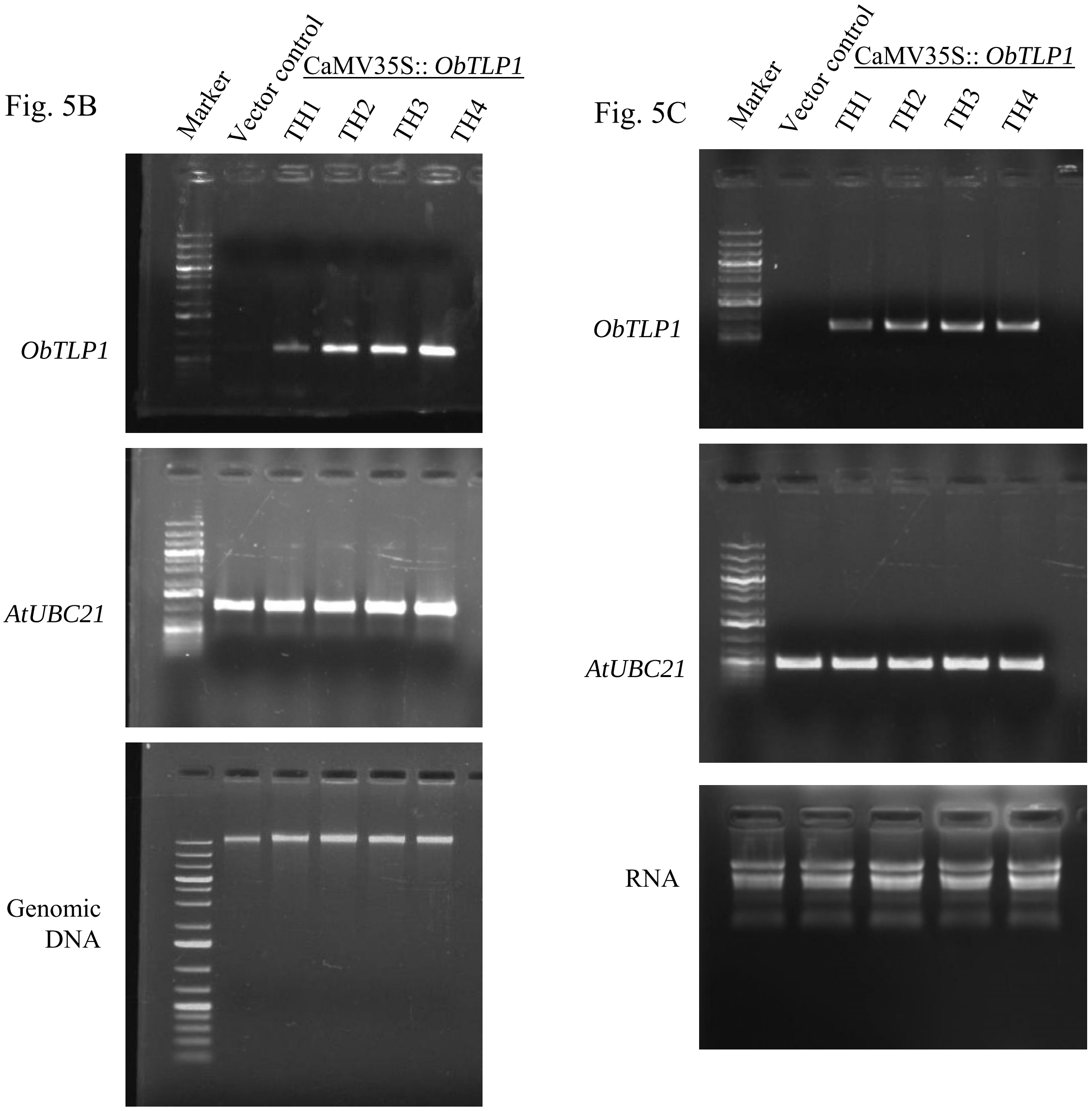

Supplementary Figure S7. Full-length gels of main figures Fig. 5B and 5C .

Supplementary Table S1. Phenotypic evaluation of *ObTLP1*-expressing Arabidopsis plants

**Parameter**	**Vector control**	**TH1**	**TH2**
Pods/plant	17.14 ± 2.38	17.79 ± 2.97	18.29 ± 2.61
Weight of mature green pod (mg)	3.13± 0.04	3.14 ± 0.04	3.12 ± 0.02
Weight of dry pod (mg)	1.12 ± 0.05	1.14 ± 0.04	1.13 ± 0.04
Seeds/pod	37.79 ± 3.72	39.5 ± 3.48	38.71 ± 3.34
100 seeds weight (mg)	2.03 ± 0.09	2.08 ± 0.13	2.06 ± 0.09

*Data are the mean ± s.d. (n=14).

Supplementary Table S2. List of the primers used in this study.

Sl. No.	Targets		Primer sequences (5′ to 3′)
			Semi-quantitative RT-PCR
1	*ObTLP1* (JQ793640)	F	ATGAATTCTCTCAAATTCA
		R	TTATGGGCAGAAGACTACC
2	*ObACT* (DY329457)	F	GCACTTCCTGTGAACAATAG
		R	AGAGGATACATGTTCACCAC
3	*AtUBC21* (AT5G25760)	F	GGCATCAAGAGCGCGACTGT
		R	GGCGAGGCGTGTATACATTT
			Quantitative RT-PCR
4	*ObTLP1 (JQ793640)*	F	AGATGACGCCGACGACTAAC
		R	CGCCGCGCACTTGAC
5	*ObEF-1α*	F	CCAATCTGACCAGGGTGGTT
		R	GACATGCGTCAGACGGTTGT
6	*ObGAPDH*	F	GGATGGCATTCCGAGTTCCT
		R	GCCCTCAGACTCCTCCTTGAT
7	*ObTIP41*	F	CGGATTCCTCATTCGTGGTT
		R	CGGCAAATGGGTTGTCTGA
8	*ObTUB*	F	CGAGCACGGAGTTGATCCA
		R	GCCGGAAGCCTCATTGAA
9	*ObUBC21*	F	CCGGTGGCCCCTTTATAAGA
		R	CGACGGCGAGGCTTTTTAA
10	*ObUBQ5*	F	GCGAAGATGAGACGCTGTTG
		R	CCTAGAGGTTGAGGCCAGTGA
11	*ObUBQ10*	F	TGGGTCTCAGTTGTCTGTTGGT
		R	CGGCAGGTGAGTTGTTACACA
12	*ObACT* (DY329457)	F	GGGCTGTTATTTCCTTGCTCAT
		R	CGTGCTCAGTGGTGGATCAA
13	*AtUBC21* (AT5G25760)	F	CTGAGCCGGACAGTCCTCTT
		R	TAGCGGCGAGGCGTGTATAC
14	*AtACT2* (AT3G18780)	F	CCTTTGTTGCTGTTGACTACGA
		R	CTGAATCTCTCAGCACCAA
15	*AtEF-1α (*AT5G60390)	F	CCCCTTCGTCTTCCACTTCA
		R	CCCTGTGGGAGCAAAGGTAA
16	*AtGAPDH (*AT1G13440)	F	CAAGGCTGCTGCTCACTTGA
		R	CGAACATGGGCGCATCTT
17	*AtTUB4 (*AT5G44340)	F	TCGGCGATTCTCCGTTACAG
		R	CAGCGCGAGGAACGTACTTT
18	*AtUBQ10 (*AT4G05320)	F	GGAGGTGGAGAGTTCTGAC
		R	GCGAAGATCAATCTCTGCT
19	*S. sclerotiorum* ITS genomic DNA (KC748491)	F	GGTGAACCTGCGGAAGGAT
		R	AAGAGCAGCTCGCCAAAGC
20	*B. cinerea Actin* genomic DNA (NW_001814525)	F	CCGTCTGGATTGGTGGTTCT
		R	CACTTGCGGTGGACAATGG
21	*AtVSP1* (AT5G24780)	F	GGGAACGAAGCCGAACTCTT
		R	GGGCACCGTGTCGAAGTTTA
22	*AtPR1* (AT2G14610)	F	CTCGGAGCTACGCAGAACAAC
		R	CCGCTACCCCAGGCTAAGTT
23	*AtPDF1* (AT5G44420)	F	CGCTGCTCTTGTTCTCTTTGC
		R	CCCTGACCATGTCCCACTTG
24	*AtPAL1* (AT2G37040)	F	CCCCTCCGTGGTACAATCAC
		R	CTTCACCGTTGGGACCAGTAG
25	*AtBGL2* (AT3G57260)	F	CCTCGACGTTCCCAGTTCAG
		R	ACTTGTCGGCCTCCGTTTG
26	*Arabidopsis Rubisco Large Subunit* (Atcg00490)	F	TGTTCTGCCTGTGGCTTCAG
		R	CCCAAGGGTGGCCTAAAGTT

			pET pET-28a(+) cloning
27	*ObTLP1* (JQ793640)	F	CTGGATCCATGAATTCTCTCAAATTCA
		R	AACTCGAGTTATGGGCAGAAGACTACC
			pBI 121 cloning
28	*ObTLP1* (JQ793640)	F	GCTCTAGAATGAATTCTCTCAAATTCATCTCC
		R	GGGAAATTTATGGGCAGAAGACTACC
